# Predicting transcriptional outcomes of novel multigene perturbations with GEARS

**DOI:** 10.1038/s41587-023-01905-6

**Published:** 2023-08-17

**Authors:** Yusuf Roohani, Kexin Huang, Jure Leskovec

**Affiliations:** 1 Department of Biomedical Data Science, Stanford University, Stanford, CA USA; 2 Department of Computer Science, Stanford University, Stanford, CA USA

**Keywords:** Gene regulatory networks, Gene expression profiling, Genomic engineering

## Abstract

Understanding cellular responses to genetic perturbation is central to numerous biomedical applications, from identifying genetic interactions involved in cancer to developing methods for regenerative medicine. However, the combinatorial explosion in the number of possible multigene perturbations severely limits experimental interrogation. Here, we present graph-enhanced gene activation and repression simulator (GEARS), a method that integrates deep learning with a knowledge graph of gene–gene relationships to predict transcriptional responses to both single and multigene perturbations using single-cell RNA-sequencing data from perturbational screens. GEARS is able to predict outcomes of perturbing combinations consisting of genes that were never experimentally perturbed. GEARS exhibited 40% higher precision than existing approaches in predicting four distinct genetic interaction subtypes in a combinatorial perturbation screen and identified the strongest interactions twice as well as prior approaches. Overall, GEARS can predict phenotypically distinct effects of multigene perturbations and thus guide the design of perturbational experiments.

## Abstract (web summary)

GEARS predicts transcriptional changes of multigene perturbations even for genes that were never experimentally perturbed.

## Main

The transcriptional response of a cell to genetic perturbation reveals fundamental insights into how the cell functions. Transcriptional responses can describe diverse functionality ranging from how gene regulatory machinery helps maintain cellular identity to how modulating gene expression can reverse disease phenotypes^1–3^. This has implications for biomedical research, especially in developing personalized therapeutics. For instance, validating drug targets through genetic perturbation studies increases the likelihood of successful clinical trials^4^. Additionally, identifying synergistic gene pairs can enhance the effectiveness of combination therapies^5–8^. Because complex cellular phenotypes are known to be produced by genetic interactions between small sets of genes, identifying such interactions could facilitate precise cell engineering^9–14^. While recent advancements have enabled scientists to more rapidly sample perturbation outcomes experimentally^9,15–19^, computational approaches that predict perturbation effects are indispensable for prioritizing experimental perturbations due to the combinatorial explosion of potential multigene combinations.

However, existing computational methods for predicting perturbational outcomes present their own limitations. The predominant approach for single-gene perturbation outcome prediction relies on inferring transcriptional relationships between genes in the form of a gene regulatory network^20–23^. This is limited either by the difficulty in accurately inferring a network from gene expression datasets^24^ or by the incompleteness of networks derived from public databases^25–27^. Moreover, existing predictive models built using such networks linearly combine the effects of individual perturbations, which renders them incapable of predicting non-additive effects of multigene perturbations, such as synergy^22^. More recent work uses deep neural networks trained on data from large perturbational screens to skip the network inference step and directly map genetic relationships into a latent space for perturbation outcome prediction^28,29^. However, these methods still require that each gene in the combination be experimentally perturbed before the effect of perturbing the combination can be predicted.

Here, we present graph-enhanced gene activation and repression simulator (GEARS), a computational method that integrates deep learning with a knowledge graph of gene–gene relationships to simulate the effects of a genetic perturbation. The incorporation of biological knowledge gives GEARS the ability to predict the outcomes of perturbing single genes or combinations of genes for which there are no prior experimental perturbation data. GEARS outperformed existing approaches in predicting the outcomes of both one-gene and two-gene perturbations drawn from seven distinct datasets. GEARS could also detect five different genetic interaction subtypes and generalize to new regions of perturbational space by predicting phenotypes that were unlike what was seen during training. Thus, GEARS can directly impact the design of future perturbational experiments.

## Results

### Knowledge-informed deep learning of perturbation effects

**Fig. 1:**
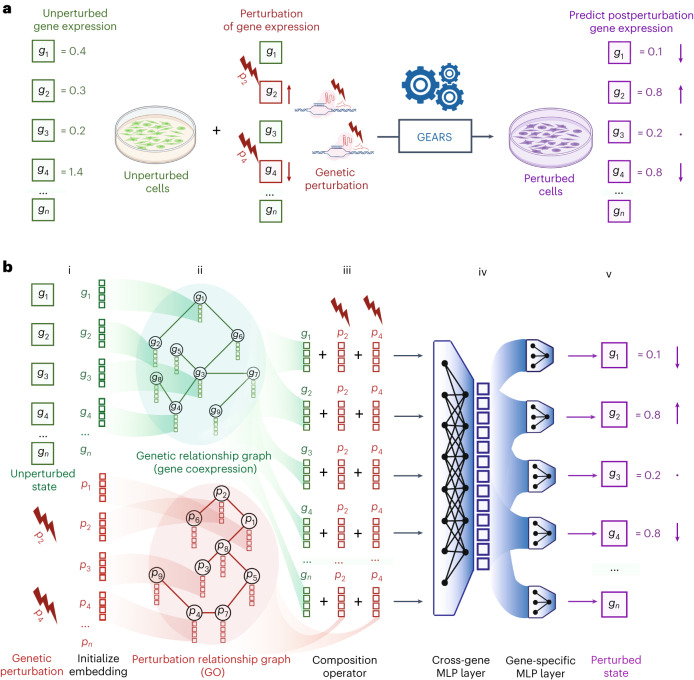
GEARS combines prior knowledge with deep learning to predict postperturbation gene expression. **a**, Problem formulation: given unperturbed gene expression (green) and applied perturbation (red), predict the gene expression outcome (purple). Each box corresponds to an individual gene. Arrows indicate change in expression. **b**, GEARS model architecture. (i) For each gene in the unperturbed state, GEARS initializes a gene embedding vector (green) and a gene perturbation embedding vector (red) (ii). These embedding vectors are assigned as node features in the gene relationship graph and the perturbation relationship graph (iii). A GNN is used to combine information between neighbors in each graph. Each resulting gene embedding is summed with the perturbation embedding of each perturbation in the perturbation set (iv). The output is combined across all genes using the cross-gene layer and fed into gene-specific output layers (v). The final result is postperturbation gene expression; MLP, multilayer perceptron.

GEARS is a deep learning-based model that predicts the gene expression outcome of combinatorially perturbing a set of one or more genes (perturbation set). Given unperturbed single-cell gene expression along with the perturbation set being applied (Fig. 1a), the output is the transcriptional state of the cell following the perturbation (Methods).

GEARS introduces a new approach of representing each gene and its perturbation using distinct multidimensional embeddings (arbitrary vectors of numbers used to represent a meaningful concept; Fig. 1b and Supplementary Note 1)^30,31^. Each gene’s embedding is tuned through the course of training to represent key traits of that gene. Splitting the representation into two multidimensional components gives GEARS additional expressivity for capturing gene-specific heterogeneity of perturbation response. Each gene’s embedding is sequentially combined with the perturbation embedding of each gene in the perturbation set and finally used to predict the postperturbation state for that gene. This prediction is conditioned on a single ‘cross-gene’ embedding vector that captures transcriptome-wide information for each cell.

GEARS is uniquely able to predict the outcomes of perturbation sets that involve one or more genes for which there are no experimental perturbation data. GEARS does this by incorporating prior knowledge of gene–gene relationships using a gene coexpression knowledge graph when learning gene embeddings and a Gene Ontology (GO)-derived knowledge graph when learning gene perturbation embeddings (Methods). This relies on two biological intuitions: (i) genes that share similar expression patterns should likely respond similarly to external perturbations, and (ii) genes that are involved in similar pathways should impact the expression of similar genes after perturbation (Fig. 1b). Different knowledge graphs, such as large context-specific networks, may prove more suitable depending on the gene set of interest^32^ (Supplementary Note 2). GEARS functionalizes this graph-based inductive bias using a graph neural network (GNN) architecture^33^.

### Predicting single-gene perturbation transcriptional responses

**Fig. 2:**
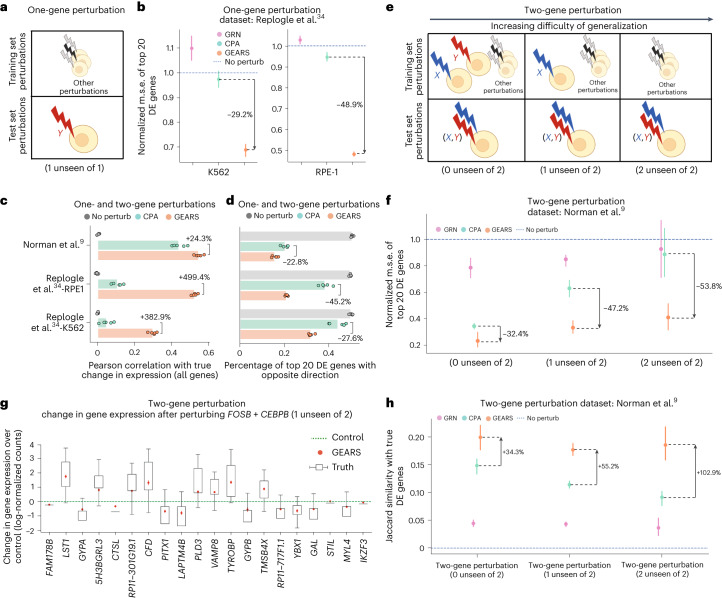
GEARS outperforms alternative approaches in predicting postperturbation gene expression. **a**, Train–test data split for single-gene perturbations. **b**, The m.s.e. in predicted postperturbation gene expression for single-gene perturbations normalized to the no perturbation case. For each perturbation, the 20 most differentially expressed (DE) genes were considered; perturb, perturbation; GRN, gene regulatory network. **c**, Pearson correlation between mean predicted postperturbation differential gene expression over control and true values across all genes. **d**, Fraction of the top 20 differentially expressed genes where the predicted postperturbation differential expression is in the opposite direction of the ground truth. **e**, Train–test data split categories for two-gene perturbations. **f**, Normalized m.s.e. in predicted postperturbation gene expression for two-gene perturbations. **g**, Boxes indicate experimentally measured differential gene expression after perturbing the gene combination *FOSB* and *CEBPB* (*n* = 85). The red symbol shows the mean change in gene expression predicted by GEARS when it has only seen *FOSB* experimentally perturbed at the time of training. The green dotted line shows mean unperturbed control gene expression. Whiskers represent the last data point within 1.5× interquartile range. **h**, Jaccard similarity between model-predicted differentially expressed genes and true differentially expressed genes. Throughout the figure, markers correspond to the mean and error bars correspond to 95% confidence intervals computed over predictions made by five models trained using different data splits (*n* = 5).

In the case of single-gene perturbations, GEARS was evaluated on the perturbation of genes whose data had been held out at the time of training, and thus those genes had not been seen experimentally perturbed during training (Fig. 2a). We used data from two different genetic perturbation screens consisting of 1,543 (RPE-1 cells) and 1,092 (K562 cells) perturbations, respectively, with each measuring over 170,000 cells (Replogle et al.^34^; Supplementary Notes 3 and 4). The screens were run using the Perturb-seq assay, which combines a pooled screen with a single-cell RNA-sequencing readout of the entire transcriptome for each cell^16^. GEARS was trained separately on each dataset. In addition to an existing deep learning-based model (CPA), we designed two alternative baseline models for evaluation of performance. One baseline model (no perturbation) assumes that the perturbation does not result in any change in gene expression. The other baseline model first infers a gene regulatory network^20^ and then linearly propagates the effects of perturbing a gene along this network (adapted from CellOracle^22^; Supplementary Notes 6 and 7).

**Extended Data Fig. 1:**
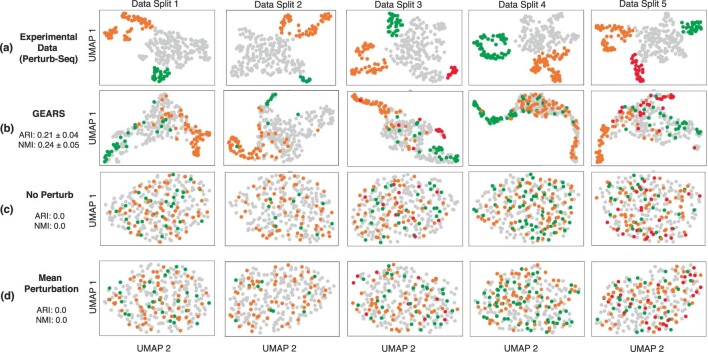
GEARS identifies groups of genes inducing similar perturbation effect, even when not seen perturbed previously. Each plot presents a low-dimensional (UMAP) representation of postperturbation gene expression following genetic perturbations that were held out in the test set. Each column corresponds to a different split of the experimental data into training and test sets. **a**, Each panel corresponds to true postperturbational transcriptional state measured using a Perturb-Seq assay. Colors correspond to distinct clusters identified using Leiden clustering set to a constant resolution across all panels. The largest cluster is assumed to show minimal perturbation effect and is colored grey. **b**, Each panel corresponds to postperturbation state predicted by GEARS. Colors correspond to the true labels identified when clustering the true experimental data, thus each point is labeled the same as in **a**. Adjusted Rand Index (ARI) and Normalized Mutual Information (NMI) were used to compare clusters identified by GEARS to those observed in true postperturbation expression for each data split. Average values for each metric across splits shown on left. **c**, Same as **b** using a baseline model that predicts no perturbation effect. **d**, Same as **b** using a baseline model that predicts mean perturbation effect.

We tested model performance by measuring the mean squared error (m.s.e.; Fig. 2b) and Pearson correlation (Fig. 2c) between the predicted postperturbation gene expression and true postperturbation expression for the held-out set (Supplementary Table 1). Because the vast majority of genes do not show substantial variation between unperturbed and perturbed states, we restricted our m.s.e. analysis to the harder task of only considering the top 20 most differentially expressed genes (Supplementary Note 8). GEARS significantly outperformed all baselines on both datasets with an m.s.e. improvement of 30–50% (Fig. 2b). When considering all genes using Pearson correlation, GEARS exhibited more than two times better performance in the case of both cell lines (Fig. 2c). Additionally, GEARS displayed a clear improvement in capturing the right direction of change in expression following perturbation (Fig. 2d), which reflects a more accurate representation of regulatory relationships. We consistently observed superior performance of GEARS over baselines across metrics (Supplementary Fig. 1) and across five additional datasets, including a genome-wide perturbation screen^16,18,34–36^ (Supplementary Table 2 and Supplementary Figs. 2 and 3). Furthermore, GEARS scaled to large datasets more effectively than conventional gene regulatory network-based methods (Supplementary Table 3). Beyond transcription levels, GEARS also identified groups of genes that induced similar transcriptional responses to perturbation, even when data for their perturbation had not been seen during training (Extended Data Fig. 1 and Supplementary Note 9).

### Predicting multigene perturbation outcomes

GEARS is designed to predict transcriptional outcomes for perturbation sets consisting of multiple genes. We evaluated performance using a Perturb-seq dataset (Norman et al.^9^) containing 131 two-gene perturbations. When evaluating GEARS on two-gene perturbations, we defined three generalization classes based on how many of the genes we see experimentally perturbed at the time of training (Fig. 2e). The first case is when the model has seen each of the two genes in the combination individually experimentally perturbed in the training data (two-gene perturbation, zero of two unseen). The other cases, which are progressively harder to predict, are when either one of the two perturbed genes (one of two unseen) or both genes (two of two unseen) have not been seen individually perturbed at the time of training (Supplementary Fig. 4 and Supplementary Note 10). GEARS improves performance by more than 30% across all cases (Fig. 2f), with the highest improvement of 53% observed when both perturbed genes in the combination are unseen. Improvements were also observed across other metrics (Supplementary Fig. 5) and on a different dataset (Supplementary Tables 2 and 4)^37^.

**Extended Data Fig. 2:**
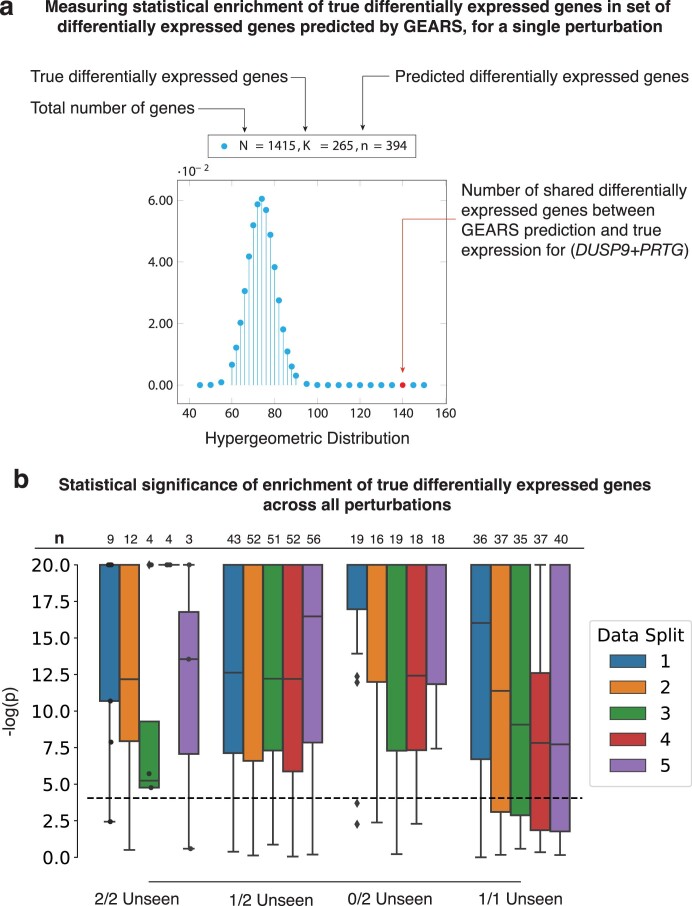
Identifying significant enrichment for true differentially expressed genes in GEARS predictions. **a**, Hypergeometric distribution used to model the probability of obtaining a random overlap between the differentially expressed genes predicted by GEARS and the true significantly differentially expressed genes following a perturbation. In this example, 142 genes were shared between GEARS and the true prediction. A p-value is calculated for each perturbation in the held out set. **b**, Box-plot showing the log (base 10) of the p-value for all held-out perturbations in the Norman et al. 2019 dataset. To account for multiple hypothesis testing (561 tests), a Bonferroni correction was applied, using a significance threshold of 0.05. A black dashed line represents the adjusted threshold. GEARS was trained on 5 different data splits (n=5). Number of data points for each bar are listed above it. Whiskers represent last data point within 1.5x interquartile range below the first quantile and above the third quantile.

Model performance was also analyzed on a gene-by-gene basis. In the case of predicting the outcome of perturbing *FOSB* with *CEBPB*, GEARS correctly captured both the right trend and the magnitude of perturbation across all 20 differentially expressed genes (Fig. 2g) even though one of the perturbed genes (*CEBPB*) had not been seen experimentally perturbed during training. Moreover, the predictions were different from the transcriptional state observed in the case of the single-gene perturbation (*FOSB*) that was seen at the time of training the model (Supplementary Fig. 6). Similar performance was observed for several other examples across generalization categories (Supplementary Fig. 7). We also measured 50% greater enrichment in the most significant differentially expressed genes as predicted by GEARS than observed with baseline methods (Fig. 2h, Extended Data Fig. 2 and Supplementary Note 11).

**Extended Data Fig. 3:**
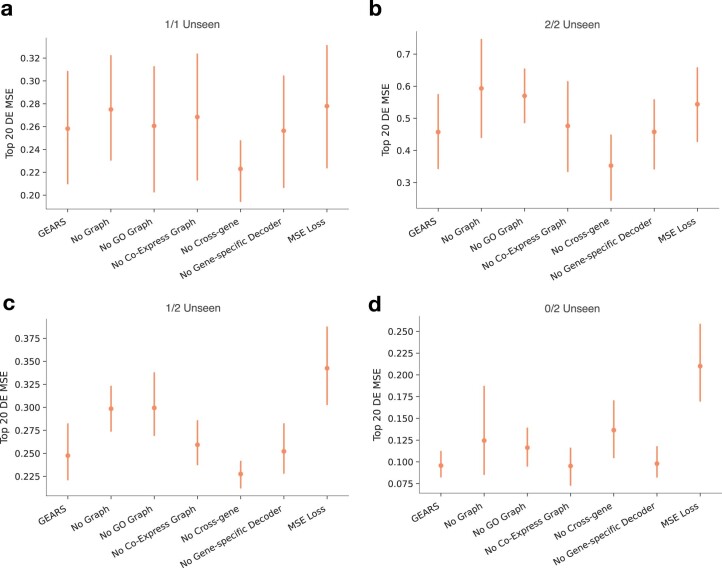
Model ablation study highlights relative importance of GEARS components under different generalization conditions. The ‘No Graph’ condition removes both the gene ontology graph and co-expression graph; ‘No GO Graph’ removes the gene ontology graph; ‘No Co-Express Graph’ removes the co-expression graph; ‘No Cross-gene’ removes the cross-gene MLP layer; ‘No Gene-specific Decoder’ removes the gene specific decoder MLP and uses a shared MLP instead; ‘MSE Loss’ switches from the auto-focus loss to the regular L2 loss. Four generalization conditions are considered: **a**, (1/1 Unseen) single-gene perturbations not seen experimentally perturbed at the time of training. **b**–**d**, (2/2 Unseen) two-gene perturbations in which both genes were not seen experimentally perturbed individually at the time of training (**b**), (1/2 Unseen) one of the two genes was not seen experimentally perturbed (**c**) or (0/2 Unseen) both genes have been seen experimentally perturbed (**d**). Performance is measured using the mean squared error in predicted postperturbation gene expression for the top 20 most differentially expressed genes. For all panels (**a**–**d**) the marker indicates the mean MSE over predictions made by models trained using 5 different training data splits (n=5). The error bars represent bootstrapped 95% CI.

**Extended Data Fig. 4:**
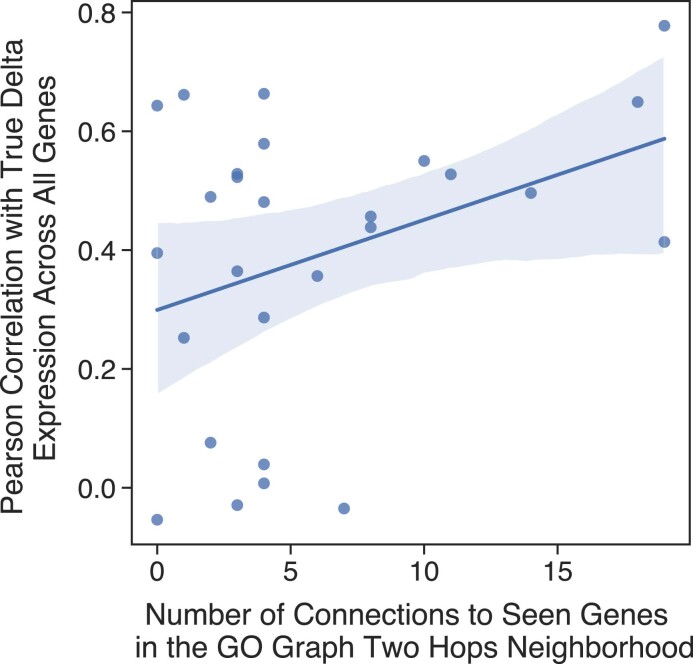
Model performance relationship with network connectivity. Each point in the scatter plot corresponds to a prediction made for a novel single-gene perturbation not seen at the time of training. The y-axis plots the pearson correlation between the true mean postperturbation differential expression over unperturbed control and the same predicted by GEARS. The x-axis measures the number of connections between the novel perturbed gene and other genes in the network that had been seen at the time of training. Error band corresponds to 95% CI.

Although the incorporation of knowledge graphs was instrumental in enabling these predictions (Extended Data Fig. 3 and Supplementary Fig. 8), it also limits the ability of GEARS to predict outcomes for perturbing previously unperturbed genes that are not well connected in this graph (Extended Data Fig. 4 and Supplementary Note 12). GEARS makes use of a Bayesian formulation to overcome this challenge by outputting an uncertainty metric that is inversely correlated with model performance (Supplementary Fig. 9).

### Predicting non-additive combinatorial perturbation effects

**Fig. 3:**
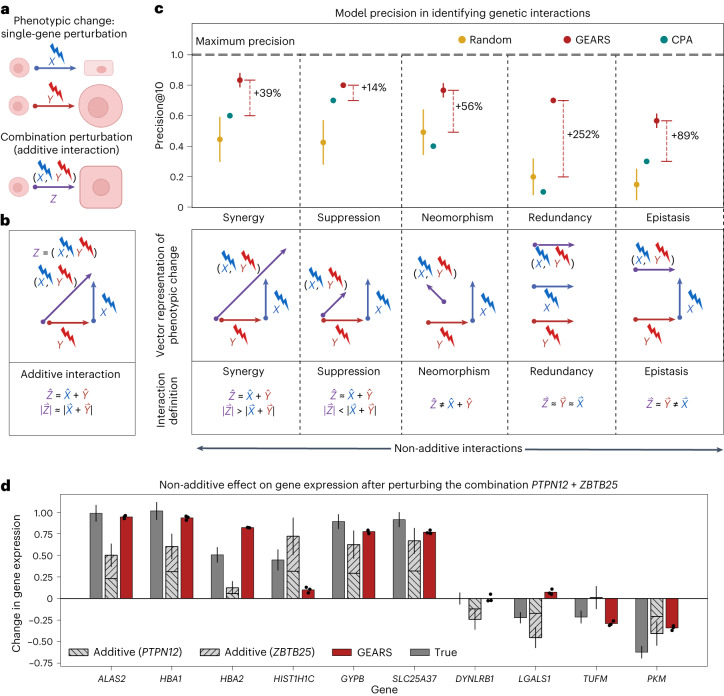
GEARS accurately predicts non-additive combinatorial effects and genetic interaction subtypes. **a**, Illustration of an additive interaction between two genes after perturbation. *X* and *Y* represent change over the unperturbed state caused by single-gene perturbations. *Z* is a combinatorial perturbation of both genes. **b**, Definition of genetic interaction subtypes. **c**, Mean precision@10 in predicting genetic interactions from 131 two-gene combinations (error bars represent s.d.). A random model performs 1,000 random draws; other models perform three predictions (*n* = 3). **d**, Change in gene expression after perturbing the combination *PTPN12* and *ZBTB25*. The gray bars show the true mean postperturbation gene expression change (*n* = 257). The hatched gray bars show the true change for each of the two single-gene perturbations performed individually (*PTPN12* *n* = 164 and *ZBTB25* *n* = 247), which are summed by the naive additive model. The red bar indicates the prediction made by GEARS (*n* = 3 trained models). Error bars correspond to 95% confidence interval.

In the case of a two-gene perturbation, if the outcomes of perturbing the two genes independently are already known, then a naive model could simply add the perturbational effects to estimate the effect of the combinatorial perturbation (Fig. 3a,b). However, genes are known to interact with one another to produce non-additive genetic interactions after perturbation. For example, two genes that independently cause a minor loss in cell growth could synergistically interact with one another following combinatorial perturbation to cause cell death.

**Extended Data Fig. 5:**
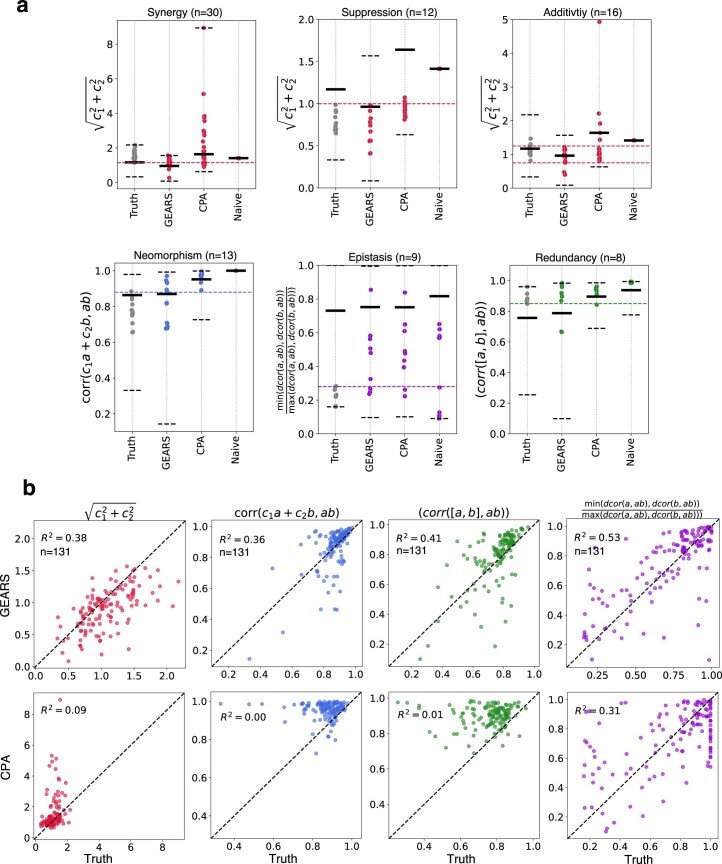
Model performance at predicting genetic interaction (GI) scores. **a**, GI scores for the set of combinatorial perturbations that were defined as expressing a specific GI subtype phenotype in Norman et al. 2019. The gray dots correspond to GI scores computed using true postperturbation gene expression. The colored dots were computed using predicted postperturbation gene expression under three different models: GEARS, CPA and Naive models. The naive model here simply sums together the effects of single-gene perturbations. The metrics on the y-axis correspond to different GI scores and the colored dotted lines indicate the defined thresholds for determining if a combination is exhibiting a specific GI subtype phenotype. Both GEARS and CPA were trained using a leave-one-out testing approach for each of the 131 combinations. The black dashed line represents the minimum and maximum of all 131 values and the black solid line represents the mean. **b**, Scatter plots of GI scores for all 131 two-gene combinatorial perturbations from that dataset. The x-axis shows GI scores computed using true postperturbation gene expression and the y-axis shows scores computed using predicted postperturbation gene expression. The top row shows predictions made by GEARS and the bottom row shows predictions made by CPA. R2 refers to the coefficient of determination.

We defined five types of genetic interactions (Supplementary Note 15): synergy, suppression, neomorphism, redundancy and epistasis (Supplementary Note 16). When both genes in a two-gene combination had been individually perturbed, the genetic interaction scores predicted by GEARS showed a stronger correlation with the ground truth scores calculated using true expression than existing methods. For instance, the correlation coefficient (*R*^2^) was approximately 0.4 for synergy, neomorphism and redundancy, whereas it was only around 0.0 for the same interactions when predicted by CPA (Extended Data Fig. 5).

**Extended Data Fig. 6:**
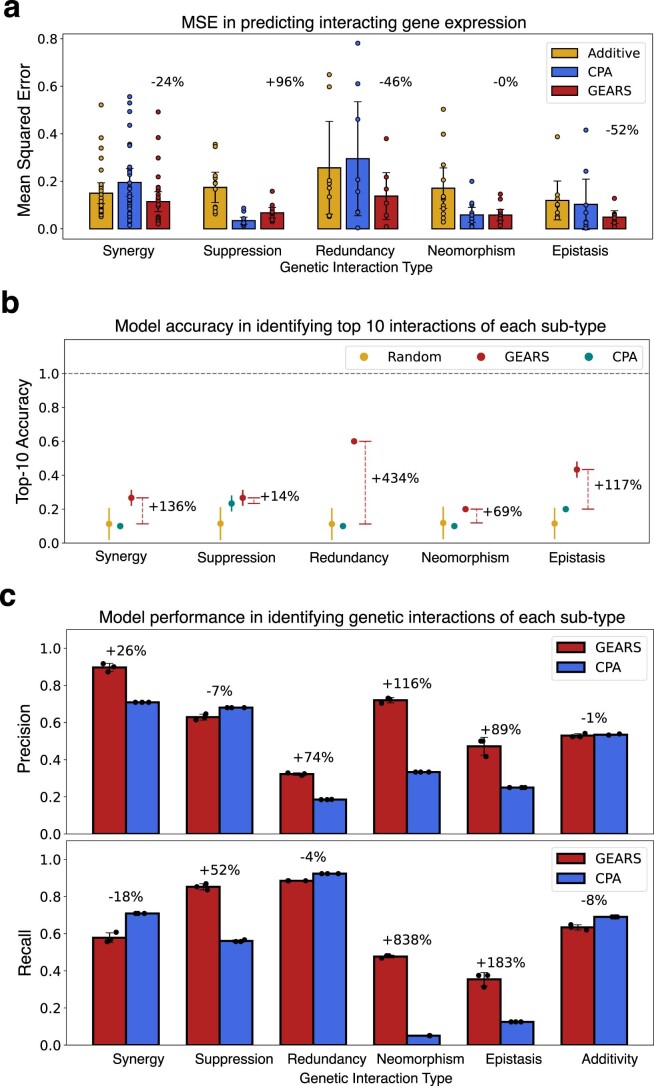
Model performance in predicting genetic interactions (GIs). **a**, Mean Square Error (MSE) in predicting non-additive combinatorial effects between the additive model which assumes that the effect of the combination is just the sum of the two known single-gene perturbation outcomes and GEARS predictions. MSE was measured on the 20 genes with the largest difference between true postperturbation expression following two-gene combinatorial perturbation and the additive prediction for that combination. GI subtypes (x-axis) were labelled without overlap as in Norman et al. 2019 (Synergy n=30, Suppression n=12, Redundancy n=8, Neomorphism n=13, Epistasis n=9). Bar plots represent the mean and error bars correspond to 95% CI. **b**, Top 10 accuracy in predicting GIs: Model accuracy in predicting the set of 10 strongest interactions for each GI subtype as determined using true expression. Marker represents mean and error bar represents 1SD for the random model which performs 1000 draws (n=1000). For other models, predictions from 3 trained models were used (n=3). **c**, Precision and recall in predicting GIs (n=3).

To identify new genetic interactions, GEARS can recommend pairs of genes that are predicted to have strong genetic interactions. To assess the real-world application of GEARS where the recommended pairs are then experimentally validated, we calculated performance metrics based on the top-ranked predictions. Precision@10 measures the fraction of predicted combinations in the top ten that truly exhibit a specific genetic interaction subtype, as determined by experimentally measured gene expression after perturbation (Supplementary Note 17). When compared to baseline methods, GEARS improved precision@10 by more than 40% for four of five genetic interaction subtypes, and the improvement exceeded 90% for redundancy and epistasis (Fig. 3c). Additionally, GEARS demonstrated a twofold increase in accuracy when predicting the ten strongest interactions for a specific genetic interaction subtype (top ten accuracy; Extended Data Fig. 6b). Further validation using an additional dataset confirmed the effectiveness of GEARS, showing a 20% increase in accuracy across four genetic interaction subtypes. Moreover, the precision–recall curves for all observed genetic interaction subtypes exhibited a higher area under the curve than other methods (Supplementary Fig. 12)^37^. In scenarios where only one gene had been perturbed previously, GEARS successfully detected synergistic and suppressive interactions (Supplementary Fig. 13).

Different types of genetic interactions can also be evaluated at the level of individual genes. For this, the 20 most affected genes were identified for each two-gene combination (Supplementary Note 18). Based on the m.s.e. for these genes, GEARS was able to capture the effects of different types of genetic interactions more than 40% better than existing methods across three of the five genetic interaction subtypes (Extended Data Fig. 6a). As an example, GEARS predicted the correct non-additive effects across almost all of the top ten non-additively expressed genes following the perturbation of *PTPN12* and *ZBTB25* (Fig. 3d). This was also observed across other examples belonging to different genetic interaction subtypes (Supplementary Fig. 14).

### Predicting new biologically meaningful phenotypes

**Fig. 4:**
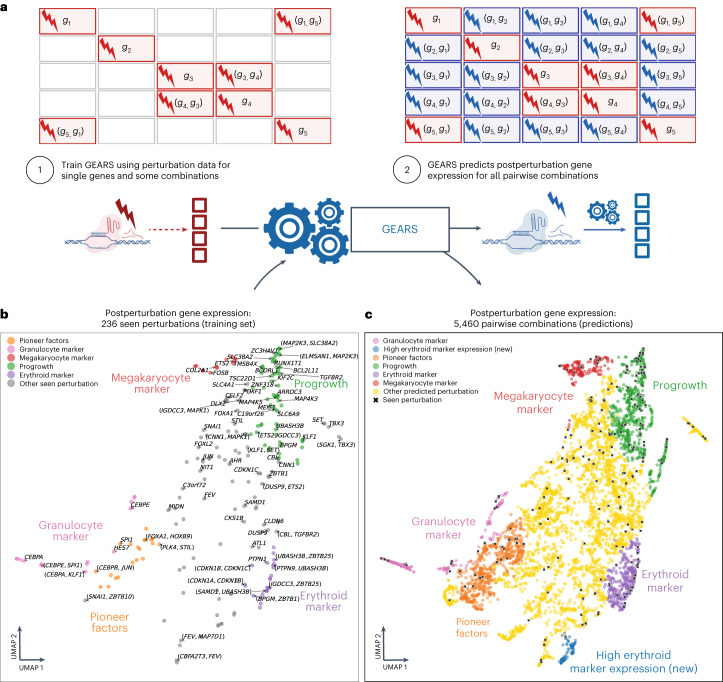
GEARS can predict new biologically meaningful phenotypes. **a**, Workflow for predicting all pairwise combinatorial perturbation outcomes of a set of genes. **b**, Low-dimensional representation of postperturbation gene expression for 102 one-gene perturbations and 128 two-gene perturbations used to train GEARS. A random selection is labeled. **c**, GEARS predicts postperturbation gene expression for all 5,151 pairwise combinations of the 102 single genes seen experimentally perturbed. Predicted postperturbation phenotypes (non-black symbols) are often different from phenotypes seen experimentally (black symbols). Colors indicate Leiden clusters labeled using marker gene expression (Supplementary Information).

We applied GEARS to the discovery of new phenotypes by predicting the outcomes of all pairwise combinatorial perturbations of 102 genes from the Norman et al. dataset^9^ (Fig. 4a). To make this prediction, GEARS was trained using the postperturbational gene expression profiles for both one-gene perturbation outcomes and 128 two-gene perturbation outcomes (Fig. 4b and Supplementary Note 13). The predicted postperturbation expression captured many distinct phenotypic clusters, including those previously identified in Norman et al.^9^ (Fig. 4c and Supplementary Note 13). Additionally, GEARS predicts a few new phenotypes, including one cluster showing high expression of erythroid markers.

To ascertain the biological relevance of this newly predicted phenotype, which was not observed in the training data, we compared it with data for proerythroblasts from the Tabula Sapiens cell atlas (Supplementary Fig. 10 and Supplementary Note 14). While this cluster’s distinct high erythroid marker expression has still not been experimentally validated, its identification demonstrates the ability of GEARS to expand the space of postperturbation phenotypes beyond what is observed in perturbational experiments. Moreover, we validated the robustness of this prediction by excluding all phenotypically similar postperturbation outcomes during training (Supplementary Fig. 11).

### Mapping combinatorial space of diverse genetic interactions

**Fig. 5:**
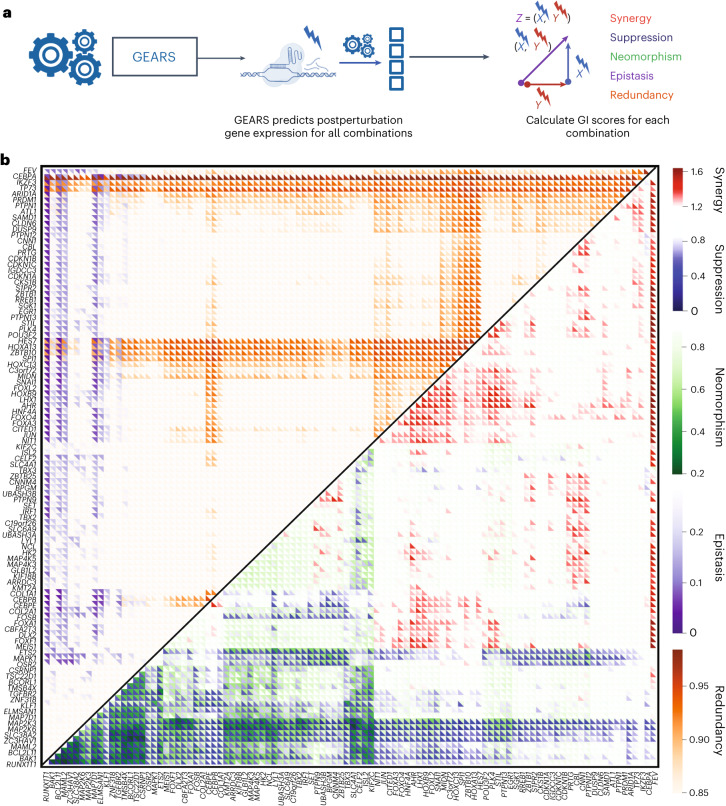
GEARS can search perturbational space for novel genetic interactions of different subtypes. **a**, Workflow for predicting genetic interaction (GI) scores. **b**, Multidimensional genetic interaction map generated by GEARS for all pairwise combinations of 102 single genes perturbed in Norman et al.^9^. For each combination, GEARS predicted genetic interaction scores for five different genetic interactions: synergy and suppression (red to blue), neomorphism (green), redundancy (orange) and epistasis (purple).

We extended our analysis to predict genetic interactions among all possible pairwise combinations of 102 genes (Fig. 5a), following CRISPRa-based combinatorial gene activation^9^. By leveraging the predicted postperturbation gene expression for each of the 5,151 pairwise combinatorial perturbations, we constructed a genetic interaction map that could simultaneously represent five distinct types of genetic interactions: synergy, suppression, neomorphism, redundancy and epistasis. The genetic interaction map revealed a rich and diverse landscape of genetic interactions, with many genes exhibiting strong tendencies toward specific genetic interaction subtypes (Fig. 5b). This effect is most evident in the interactions between functionally related genes, which is in line with previous experimental results^15,16,38^. For instance, genes involved in early erythroid differentiation pathways (*PTPN12*, *IKZF3* and *LHX1*) show a consistent trend of strong synergistic interactions with one another. Moreover, the uniqueness of this genetic interaction map is in how it captures a much broader range of interactions than a conventional genetic interaction map, which focuses primarily on synergistic or buffering interactions (Supplementary Fig. 15)^15^.

**Extended Data Fig. 7:**
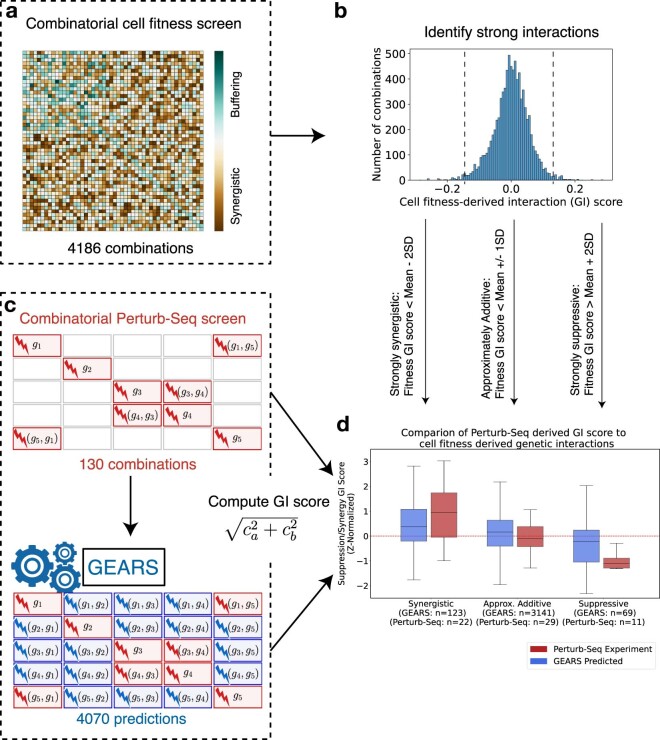
Validation of GEARS predicted genetic interaction (GI) map using combinatorial cell fitness screen. **a**, Combinatorial cell fitness screen data was used for all pairwise combination of 92 genes leading to 4186 unique combinations. Using cell fitness, interactions were quantified as synergistic or suppressive. **b**, Combinations showing the strongest cell fitness effects were used to validate GEARS predictions. **c**, Combinatorial Perturb-seq data was available for 110 of these combinations. GEARS was trained on Perturb-Seq data to predict remaining 4076 perturbation outcomes. **d**, GEARS performs similar to experimental Perturb-Seq data in predicting strong genetic interaction outcomes for both strongly synergistic and suppressive interactions identified using cell fitness measurements. GI scores are z-normalized within each modality for comparison. Centreline represents mean. Whiskers represent last data point within 1.5x interquartile range below the first quantile and above the third quantile, outliers not shown. The p-values were computed using a one-sided t-test comparing the means of the two distributions.

To validate some of these predictions, we used data from a cell fitness screen that perturbed all pairwise combinations of 92 genes^9^ (Supplementary Note 19). GEARS performed comparably to a real Perturb-seq experiment in capturing the strong interaction effects observed in the cell fitness screen (Extended Data Fig. 7). The distribution of GEARS-predicted genetic interaction scores was significantly higher for perturbations showing synergistic cell fitness effects (*P* < 0.0013, *n* = 123; data were analyzed by one-sided *t*-test comparing the means) and lower for those showing buffering effects (*P* < 4 × 10^−5^, *n* = 69) than those showing approximately additive cell fitness effects. These findings increase our confidence that several strong interactions captured in the genetic interaction map are biologically meaningful even though not all predictions have been experimentally validated. When trained to directly predict cell fitness, GEARS also showed strong performance (*R*^2^ between 0.64 and 0.93; Supplementary Figs. 16 and 17 and Supplementary Note 20).

## Discussion

Recent advancements in high-throughput perturbational screens have enhanced both the precision with which genes can be targeted^39,40^ and the scale of information generated^17,34^. However, their scalability is limited due to cost. As CRISPR-based perturbational screens become more widely used in drug discovery, GEARS can serve as a valuable complement to these experiments. GEARS has the unique ability to infer a broader range of multigene perturbation outcomes using the same experimental data as existing methods^19,41^. Furthermore, GEARS can guide the design of new screens by identifying perturbations that maximize information gained and minimize experimental costs (Extended Data Fig. 4).

However, for reliable predictions, GEARS must be trained on the same cell type or experimental condition. Moreover, training GEARS using combinatorial perturbation data is essential for accurate prediction of multigene perturbations. Various confounding factors in the data can also influence the accuracy of predictions, including cell cycle effects, the assumed success of gene editing experiments and heterogeneity in postperturbation distribution (Supplementary Note 21).

One of the important strengths of GEARS is detecting emergent interactions between pairs of genes. This feature enhances the discovery of feasible routes for engineering cell identity, where cells are guided between transcriptional states that may be significantly different from one another. For example, GEARS can aid in the precise reengineering of immune cells to prevent exhaustion when targeting cancer^14,42^ or in the reversal of phenotypes linked to aging^43–45^. Moreover, models like GEARS could predict effective cocktails of transcription factors for reprogramming induced pluripotent stem cells into individual-specific in vitro models^46–50^. Therefore, GEARS holds promise to not only impact the discovery of novel small molecules for targeting disease but also aid in designing the next generation of cell- and gene-based therapeutics.

## Methods

### Overview of GEARS

GEARS considers a perturbation dataset of *N* cells ${{{\mathcal{D}}}}={\{({{{{\bf{g}}}}}^{i},{{{{\mathcal{P}}}}}^{i})\}}_{i = 1}^{N}$, where ${{{{\bf{g}}}}}^{i}\in {{\mathbb{R}}}^{K}$ is the gene expression vector of cell *i* with *K* genes, and ${{{{\mathcal{P}}}}}^{i}=({P}_{1}^{i},\cdots \,,{P}_{M}^{i})$ is the set of perturbations of size *M* performed on cell *i*. *M* = 0 corresponds to an unperturbed cell. Each perturbation *P*_*k*_ in the set corresponds to the index of a gene. The goal of GEARS is to learn a function *f* that maps a novel perturbation set ${{{\mathcal{P}}}}$ to its postperturbation outcome, which is a gene expression vector **g**.

Specifically, given a perturbation set ${{{\mathcal{P}}}}=({P}_{1},\cdots \,,{P}_{M})$, GEARS first applies a GNN encoder ${f}_{{{\mbox{pert}}}}:{\mathbb{Z}}\longrightarrow {{\mathbb{R}}}^{d}$ that maps each genetic perturbation $P\in {{{\mathcal{P}}}}$ to a *d*-dimensional gene perturbation embedding. Another GNN-based encoder ${f}_{{{\mbox{gene}}}}:{\mathbb{Z}}\longrightarrow {{\mathbb{R}}}^{d}$ maps each gene into a gene embedding. GEARS then combines the set of perturbation embeddings with each of the gene embeddings using a compositional module. A cross-gene decoder ${f}_{{{\mbox{dec}}}}:{\{{{\mathbb{R}}}_{i}^{d}\}}_{i = 1}^{K}\longrightarrow {{\mathbb{R}}}^{K}$ then takes in the set of perturbed gene embeddings and maps them to the postperturbation gene expression vector. The entire network is trained end to end with an autofocus direction-aware loss (Supplementary Note 22).

### Gene coexpression graph encoder

To capture the relative heterogeneity of perturbational response for each gene, GEARS represents each gene $u\in {\mathbb{Z}}$ as a learnable embedding ${{{{\bf{x}}}}}^{{{\mbox{gene}}}}\in {{\mathbb{R}}}^{d}$ instead of a scalar. GEARS first obtains a representation for each gene that captures coexpression patterns in the cell. For this, we apply a GNN on a gene coexpression graph ${{{{\mathcal{G}}}}}_{{{\mbox{gene}}}}$, where edges link coexpressed genes (nodes). GEARS calculates Pearson correlations *ρ*_*u*,*v*_ among genes *u*,*v* in the training dataset. For each gene *u*, we connect it to the top *H*_gene_ genes that have the highest *ρ*_*u*,*v*_ and are above a threshold *δ*. Next, we apply a GNN parameterized by *θ*_*g*_ that augments every gene *u*’s embedding ${{{{\bf{x}}}}}_{u}^{\,{{\mbox{gene}}}\,}$ by integrating information from the embeddings of its coexpressed genes: ${{{{\bf{h}}}}}_{u}^{\,{{\mbox{gene}}}\,}={{{{\rm{GNN}}}}}_{{\theta }_{g}}\left({{{{\bf{x}}}}}_{u}^{\,{{\mbox{gene}}}\,},{{{{\mathcal{G}}}}}_{{{\mbox{gene}}}}\right)\in {{\mathbb{R}}}^{d}$.

### Incorporating prior knowledge of gene–gene relationships using the GO graph

GEARS predicts the outcome of perturbing genes never seen perturbed before by constructing a gene perturbation similarity graph ${{{{\mathcal{G}}}}}_{{{\mbox{pert}}}}$, leveraging the pathway information contained in GO^51^. We first define ${{{{\mathcal{G}}}}}_{{{\mbox{GO}}}}$ as a bipartite graph where an edge links a gene to a pathway GO term. We denote ${{{{\mathcal{N}}}}}_{u}$ as the set of pathways for a gene *u*. We compute the Jaccard index between a pair of genes *u*,*v* as ${J}_{u,v}=\frac{| {{{{\mathcal{N}}}}}_{u}\cap {{{{\mathcal{N}}}}}_{v}| }{| {{{{\mathcal{N}}}}}_{u}\cup {{{{\mathcal{N}}}}}_{v}| }$; this measures the fraction of shared pathways between the two genes. For each gene *u*, we then select the top *H*_pert_ gene *v* with the highest *J*_*u*,*v*_ to construct ${{{{\mathcal{G}}}}}_{{{\mbox{pert}}}}$. Next, we initialize all possible gene perturbations (*P*_1_,⋯,*P*_*K*_) with learnable embeddings $({{{{\bf{x}}}}}_{1}^{\,{{\mbox{pert}}}\,},\cdots \,,{{{{\bf{x}}}}}_{K}^{\,{{\mbox{pert}}}\,})$. We then feed them into a GNN parameterized by *θ*_*p*_ to augment every perturbation *v*’s embedding ${{{{\bf{x}}}}}_{v}^{\,{{\mbox{pert}}}\,}$ by integrating information from neighboring perturbations in ${{{{\mathcal{G}}}}}_{{{\mbox{pert}}}}$: ${{{{\bf{h}}}}}_{v}^{\,{{\mbox{pert}}}\,}={{{{\rm{GNN}}}}}_{{\theta }_{p}}({{{{\bf{x}}}}}_{v}^{\,{{\mbox{pert}}}\,},{{{{\mathcal{G}}}}}_{{{\mbox{pert}}}})\in {{\mathbb{R}}}^{d}$.

### Modeling combinatorial perturbations across genes

Given a perturbation set ${{{\mathcal{P}}}}=({P}_{1},\cdots \,,{P}_{M})$, GEARS looks up the perturbation embedding of each element of that set $({{{{\bf{h}}}}}_{{P}_{1}}^{\,{{\mbox{pert}}}\,},\cdots \,,{{{{\bf{h}}}}}_{{P}_{M}}^{\,{{\mbox{pert}}}\,})$. To model multigene perturbations, we use the ‘sum’ compositional operator followed by an MLP: ${{{{\bf{h}}}}}^{{{{\mathcal{P}}}}}={{{{\rm{MLP}}}}}_{{\theta }_{c}}\left(\mathop{\sum }\nolimits_{i = 1}^{M}{{{{\bf{h}}}}}_{{P}_{i}}^{\,{{\mbox{pert}}}\,}\right)$. The ‘sum’ operator allows extendability to perturbations of any size. Thus, each perturbation embedding from $({{{{\bf{h}}}}}_{{P}_{1}}^{\,{{\mbox{pert}}}\,},\cdots \,,{{{{\bf{h}}}}}_{{P}_{M}}^{\,{{\mbox{pert}}}\,})$ is applied to every gene embedding to obtain a postperturbation gene embedding. For gene *u*, we have ${{{{\bf{h}}}}}_{u}^{\,{{\mbox{post-pert}}}\,}={{{{\rm{MLP}}}}}_{{\theta }_{pp}}\left({{{{\bf{h}}}}}_{u}^{\,{{\mbox{gene}}}\,}+{{{{\bf{h}}}}}^{{{{\mathcal{P}}}}}\right)$.

### Cross-gene effects and gene-specific decoder

Following application of the perturbations in the embedding space, GEARS maps the postperturbation gene embedding to its corresponding postperturbation gene expression vector. Because each gene has its own perturbation pattern, for every gene *u*, we apply a gene-specific linear layer parameterized by ${{{{\bf{w}}}}}_{u}\in {{\mathbb{R}}}^{d},{b}_{u}\in {\mathbb{R}}$ to map it to a scalar of perturbation gene expression effect ${{{{\bf{z}}}}}_{u}={{{{\bf{w}}}}}_{u}{{{{\bf{h}}}}}_{u}^{\,{{\mbox{post-pert}}}\,}+{b}_{u}\in {\mathbb{R}}$. We then concatenate the individual effect to a single perturbation effect vector ${{{\bf{z}}}}\in {{\mathbb{R}}}^{K}$ for the cell. Because the perturbational effect on a gene can incur secondary effects on other genes, we wanted to use the transcriptome-wide ‘cross-gene’ information for the cell when predicting final gene expression for each gene. Thus, we added an additional MLP that generates a cross-gene embedding for the cell ${{{{\bf{h}}}}}^{{{\mbox{cg}}}}={{{{\rm{MLP}}}}}_{{\theta }_{cg}}\left({{{\bf{z}}}}\right)\in {{\mathbb{R}}}^{d}$. Conditioned on this cross-gene state, for every gene *u*, a gene-specific decoder parameterized by ${{{{\bf{w}}}}}_{u}^{\,{{\mbox{cg}}}\,}\in {{\mathbb{R}}}^{d+1},{b}_{u}^{\,{{\mbox{cg}}}\,}\in {\mathbb{R}}$ augments **z**_*u*_ to ${\hat{{{{\bf{z}}}}}}_{u}={{{{\bf{w}}}}}_{u}^{\,{{\mbox{cg}}}\,}\left({{{{\bf{z}}}}}_{u}\parallel {{{{\bf{h}}}}}^{{{\mbox{cg}}}}\right)+{b}_{u}^{{{\mbox{cg}}}\,}\in {\mathbb{R}}$, where the double bar notation (∥) refers to the vector concatenation operation. Finally, the predicted perturbation effect vector $\hat{{{{\bf{z}}}}}\in {{\mathbb{R}}}^{K}$ is added to the gene expression of a randomly sampled unperturbed control cell (**g**_ctrl_) to arrive at the predicted postperturbation gene expression vector for that cell $\hat{{{{\bf{g}}}}}=\hat{{{{\bf{z}}}}}+{{{{\bf{g}}}}}_{{{{\rm{ctrl}}}}}$. This allows GEARS to focus only on learning perturbation effects.

### Autofocus direction-aware loss

GEARS optimizes model parameters to fit the predicted $\hat{{{{\bf{g}}}}}$ postperturbation gene expression to true postperturbation gene expression **g** using stochastic gradient descent. We designed an autofocus loss that automatically gives a higher weight to differentially expressed genes by elevating the exponent of the error. Given a minibatch of *T* perturbations, where each perturbation *k* has *T*_*k*_ cells and each cell has *K* genes with predicted postperturbation gene expression $\hat{{{{\bf{g}}}}}$ and true expression **g**, the loss is defined as

$${L}_{{{{\rm{autofocus}}}}}=\frac{1}{T}\mathop{\sum }\limits_{k=1}^{T}\frac{1}{{T}_{k}}\mathop{\sum }\limits_{l=1}^{{T}_{k}}\frac{1}{K}\mathop{\sum }\limits_{u=1}^{K}{({{{{\bf{g}}}}}_{u}-{\hat{{{{\bf{g}}}}}}_{u})}^{(2+\gamma )}.$$

However, this loss is insensitive to directionality. To address this, GEARS incorporates an additional direction-aware loss

$${L}_{{{{\rm{direction}}}}}=\frac{1}{T}\mathop{\sum }\limits_{k=1}^{T}\frac{1}{{T}_{k}}\mathop{\sum }\limits_{l=1}^{{T}_{k}}\frac{1}{G}\mathop{\sum }\limits_{u=1}^{K}{\left[{{{\rm{sign}}}}\left({{{{\bf{g}}}}}_{u}-{{{{\bf{g}}}}}_{u}^{{{{\rm{ctrl}}}}}\right)-{{{\rm{sign}}}}\left({\hat{{{{\bf{g}}}}}}_{u}-{{{{\bf{g}}}}}_{u}^{{{{\rm{ctrl}}}}}\right)\right]}^{2}.$$

The prediction loss function is *L* = *L*_autofocus_ + *λL*_direction_, where *λ* adjusts the weight for the directionality loss.

### Uncertainty

GEARS generates an uncertainty score to measure the confidence of model prediction on a novel perturbation. A Gaussian likelihood ${{{\mathcal{N}}}}({\hat{{{{\bf{g}}}}}}_{u},{\hat{\sigma }}_{u}^{2})$ is used to model the postperturbation gene expression value for gene *u* under perturbation ${{{\mathcal{P}}}}$, where ${\hat{{{{\bf{g}}}}}}_{u}$ is the predicted postperturbation scalar and ${\hat{\sigma }}_{u}^{2}$ is the variance^52^. We add an additional gene-specific layer to predict the log variance term ${s}_{u}=\log {\hat{\sigma }}_{u}^{2}={{{{\bf{w}}}}}_{u}^{{{{\rm{unc}}}}}{{{{\bf{h}}}}}_{u}^{\,{{\mbox{post-pert}}}}+{b}_{u}^{{{\mbox{unc}}}\,}$ for each gene *u* and learn it through a modified Bayesian neural network loss^52^

$${L}_{{{{\rm{unc}}}}}=\frac{1}{T}\mathop{\sum }\limits_{k=1}^{T}\frac{1}{{T}_{k}}\mathop{\sum }\limits_{l=1}^{{T}_{k}}\frac{1}{G}\mathop{\sum }\limits_{u=1}^{K}\exp (-{s}_{u}){({{{{\bf{g}}}}}_{u}-{\hat{{{{\bf{g}}}}}}_{u})}^{(2+\gamma )}.$$

By encouraging log variance to be large when the error is large, the log variance is learned to be a proxy of model uncertainty.

### Reporting summary

Further information on research design is available in the Nature Portfolio Reporting Summary linked to this article.

## Online content

Any methods, additional references, Nature Portfolio reporting summaries, source data, extended data, supplementary information, acknowledgements, peer review information; details of author contributions and competing interests; and statements of data and code availability are available at 10.1038/s41587-023-01905-6.

### Supplementary information

Supplementary Information Supplementary Notes 1–23, Tables 1–6 and Figs. 1–20.

Reporting Summary

## Data Availability

The following are the Gene Expression Omnibus accession numbers used: Dixit et al.^16^: GSE90063; Adamson et al.^18^: GSE90546; Norman et al.^9^: GSE133344; Jost et al.^35^: GSE132080; Tian et al.^36^: GSE124703; Replogle et al.^37^: GSE146194; Horlbeck et al.^15^: GSE116198. The data from Replogle et al.^34^ are available at 10.25452/figshare.plus.20022944.

## Code availability

Code to run GEARS is available at https://github.com/snap-stanford/GEARS. Results can be reproduced using https://github.com/yhr91/GEARS_misc.
