# Supplementary material for: Predicting transcriptional outcomes of novel multigene perturbations with GEARS
Source: Nat Biotechnol. 2023 Aug 17;42(6):927–35. doi: 10.1038/s41587-023-01905-6 (PMC11180609; doi:10.1038/s41587-023-01905-6)
Supplement: Supplementary file 1 — Supplementary Notes 1–23, Tables 1–6 and Figs. 1–20. [file 41587_2023_1905_MOESM1_ESM.pdf]

nature biotechnology

Article

https://doi.org/10.1038/s41587-023-01905-6

Predicting transcriptional outcomes of novel multigene perturbations with GEARS

In the format provided by the authors and unedited

Supplementary Notes

1. **Supplementary Note 1**: Embeddings
2. **Supplementary Note 2**: Choice of graph to represent prior knowledge
3. **Supplementary Note 3**: Data preprocessing
4. **Supplementary Note 4**: Selection of strong perturbations
5. **Supplementary Note 5**: Baseline models
6. **Supplementary Note 6**: GRN-inference methods and their applicability to multi-gene perturbation outcome prediction
7. **Supplementary Note 7**: Designing a gene regulatory network (GRN) baseline and leveraging existing models
8. **Supplementary Note 8**: Identification of most significant differentially expressed genes
9. **Supplementary Note 9**: Identification of known clusters of post-perturbation transcriptional effect within previously unseen perturbations
10. **Supplementary Note 10**: Generating a data split for model evaluation
11. **Supplementary Note 11**: Comparison of differentially expressed genes across models
12. **Supplementary Note 12**: Model ablation analysis across generalization conditions
13. **Supplementary Note 13**: Generating UMAPs and clustering of post-perturbation gene expression data
14. **Supplementary Note 14**: Differential expression analysis for novel phenotypic cluster identified by GEARS
15. **Supplementary Note 15**: Measuring genetic interaction scores
16. **Supplementary Note 16**: Identifying genetic interaction subtypes
17. **Supplementary Note 17**: Model evaluation for predicting genetic interactions

18. **Supplementary Note 18**: Model evaluation for predicting non-additive effects

19. **Supplementary Note 19**: Validation of genetic interaction map using combinatorial cell fitness screen

20. **Supplementary Note 20**: Predicting endpoint phenotypic effects

21. **Supplementary Note 21**: Analysis of confounding factors

22. **Supplementary Note 22**: Hyperparameter search

23. **Supplementary Note 23**: Selecting predictions with low uncertainty

Supplementary Tables

- **Supplementary Table 1**: Performance Metrics
- **Supplementary Table 2**: GEARS outperforms baselines across multiple datasets
- **Supplementary Table 3**: Scalability of GEARS across different datasets.
- **Supplementary Table 4**: Performance improvement across generalization conditions for a 2-gene perturbation dataset
- **Supplementary Table 5**: Performance comparison of different network inference based baselines
- **Supplementary Table 6**: Performance comparison of different modifications to CPA using a knowledge graph

Supplementary Figures

- **Supplementary Fig. 1**: Comprehensive evaluation establishes robustness of GEARS's prediction of post-perturbation expression)
- **Supplementary Fig. 2**: GEARS shows consistent improvement in performance over baselines across 7 datasets with diverse cell types, number of perturbations and experimental conditions (Pearson Correlation)
- **Supplementary Fig. 3**: GEARS shows consistent improvement in performance over baselines across 7 datasets with diverse cell types, number of perturbations and experimental conditions (MSE)
- **Supplementary Fig. 4**: Data split matrix
- **Supplementary Fig. 5**: Subgroup analysis of additional evaluation metrics for predicting post-perturbation expression
- **Supplementary Fig. 6**: GEARS predicted post-perturbation gene expression for 2-gene combination is different from the previously seen 1-gene perturbation phenotype
- **Supplementary Fig. 7**: Examples of predicted gene expression across 20 most differentially expressed genes after combinatorial perturbation
- **Supplementary Fig. 8**: Model Ablation performance across metrics
- **Supplementary Fig. 9**: Variation in model performance between predictions with low uncertainty and others
- **Supplementary Fig. 10**: GEARS-predicted phenotype is biologically meaningful
- **Supplementary Fig. 11**: Prediction of novel phenotype robust to witholding of phenotypically similar data points
- **Supplementary Fig. 12**: Performance evaluation of GEARS on predicting genetic interactions in Replogle et al. 2020

- **Supplementary Fig. 13**: Model performance at predicting GI scores when one of the genes in a combination has not been experimentally perturbed
- **Supplementary Fig. 14**: GEARS predicts non-additive combinatorial effects across GI subtypes.
- **Supplementary Fig. 15**: Illustration of how multi-dimensional GI map can capture significant differences in transcriptional response.
- **Supplementary Fig. 16**: GEARS architecture can be repurposed for predicting endpoint phenotypic effects
- **Supplementary Fig. 17**: GEARS accurately predicts cell fitness scores following combinatorial perturbation
- **Supplementary Fig. 18**: Relationship between word embeddings in NLP models and gene perturbation embeddings in GEARS
- **Supplementary Fig. 19**: Impact of varying prior knowledge on GEARS' performance
- **Supplementary Fig. 20**: Performance comparison of GRN-Baseline, CellOracle and GEARS

Supplementary Notes

Supplementary Note 1: Embeddings.

- **What are embeddings and how are they used in machine learning models?** An embedding can be thought of an arbitrarily shaped vector of numbers that represents a meaningful concept. An embedding is a useful tool because it can easily be manipulated by a machine learning model to represent that concept most effectively, as measured by some optimization function that is used to train the model. For instance, an image embedding can be continuously tuned until it can effectively differentiate between different classes of objects such as cats and dogs. Once it does so effectively, a new image could be quickly mapped to this space to make this classification.

  GEARS initializes embeddings for each gene randomly and tunes them over the course of training to capture a meaningful representation of that gene. These multi-dimensional gene embeddings enable better modeling of perturbations and better generalization to previously unseen genes. We go one step further and separate the concept of a gene into two parts, one representation (embedding) corresponding to the perturbation of that gene and the other representing the expression of that gene.

- **How GEARS also uses a latent space to predict non-additive effects:**

  Let's first consider the example of word embeddings in the Natural Language Processing (NLP) literature. Similar to our formulation in GEARS, NLP models will often represent words using embeddings or multi-dimensional vectors in a latent space. The goal for the machine learning (ML) model is to learn the best mapping function f_e from input word to an embedding such that some defined operation can be performed most optimally. For instance, here we see the representation for four words "King", "Queen", "Man" and "Woman" using four vectors respectively: $h_{King} = f_e(King)$, $h_{Queen} = f_e(Queen)$, $h_{Man} = f_e(Man)$, $h_{Woman} = f_e(Woman)$. A well-trained model would be able to use vector alegbra to make novel inferences in the latent space. For instance, the ML model may learn to tune f_e such that the vector $p = h_{Woman} - h_{Man}$ (solid red arrow) could be used to bring about a change

from male to female for any entity in the latent space (Supplementary Figure 18a). So, if we then have some representation of the idea of male royalty using h_{King}, then even if the model wasn't explicitly trained with this relational information (say it was held out), then it could still answer what word is used for female royalty: i.e. $h_{King} + p = h_{Queen}$. This operation is non-trivial because no amount of adding or concatenating of text strings would have lead to this discovery, it was only possible through the learning of some meaningful representation of these words.

Similar to the example above we can also abstract genes using embeddings. Consider two mapping functions f_e and f_p which correspond to the gene embedding mapping and the gene perturbation embedding mapping function in GEARS. Consider some genes g_i with gene embedding h_i and some genes g_j, g_k with perturbation embeddings p_j and p_k. Let's say that GEARS has learnt from the training data that if any perturbation brings a gene embedding vector to an orientation that points below the x-axis (negative slope) then it leads to a non-additive suppressive effect on that gene. Thus, we can see from the figure that even though single-gene perturbations p_j and p_k did not cause the suppressive effect to activate, both of them combined did so ($p_j + p_k$) (Supplementary Figure 18b). Thus, using a latent space GEARS has more flexibility to generalize to new non-additive effects that are not seen in the data.

Supplementary Note 2: Choice of graph to represent prior knowledge.

GEARS does not require a specific representation of prior knowledge about gene-gene relationships. We experimented with a few different networks to use in place of the Gene Ontology network including a protein-protein interaction network [1], a gene coessentiality network [2] and a gene co-expression network. We decided to proceed with the Gene Ontology network because it had the best coverage over the gene set that we were interested in, produced very good predictive performance and was the most general-purpose for application to future tasks.

We also studied the impact of varying the components of the Gene Ontology graph that are used as prior knowledge as well as the effect of cell-type specific versus cell-type agnostic prior knowledge (Supplementary Figure 19). Data from the Replogle et al. 2022 [3] dataset for the RPE-1 (E) cell line was used for this analysis (Supplementary Note 4).

- **Varying Gene Ontology components**: We first studied the impact of varying the components of the Gene Ontology graph used as background knowledge for the perturbation embeddings. This results in minimal impact on performance with a slight improvement in case of biological process alone. This fits with our original assumption behind using GO, which is that genes that share similar biological pathways should result in similar perturbation effects. While molecular function is often overlapping with biological process annotation, biological process is more diverse and represents specific biological ending states or outcomes [4].

- **Varying resolution of GO graph**: The Gene Ontology graph is generated by identifying the top-k genes with most similar annotation to a given source gene (as measured using the Jaccard similarity, see Methods). These k genes are then connected to the source gene in the GO graph. Thus, by varying the k parameter, we can influence the density of edges in the graph. Upon reducing the value of k and thus the density of the graph, we observe that the performance of GEARS decreases.

- **Varying Gene Coexpression Network**: We tested the influence of varying the gene coexpression network on predictive performance. Since the gene co-expression graph is inferred using expression data for the cell type of interest, this also allowed us to study the impact of varying cell-type specific prior knowledge. We make two observation based on the results.

  Performance appears to be consistent across three other cell types from four different datasets, both when considered individually and altogether. This would imply that the information added through using the gene co-expression graph is invariant to the cell type used to infer the graph. Taking into account previous results from the model ablation study where the influence of removing the gene co-expression graph was limited, we hypothesize that the value added by this graph is probably dominated by a few strongly co-expressed genes that occur across cell types.

  We also observe that coexpression information from other cell types appears to outperform that for the cell type for which GEARS was making predictions (RPE-1). While this appears counter-intuitive, it reflects the challenges of correctly inferring a coexpression network using gene expression data. The graph inferred using RPE-1 data is likely noisier even if

more biologically relevant. The challenge of correctly inferring a coexpression network is also evident in the larger error bars and poorer performance observed for larger datasets in this analysis. For instance, even when considering the same cell type K562, the error bars are wider when inferring the coexpression network using genome-wide perturbation dataset consisting of 2 million cells as compared to the essential perturbation dataset consisting of 250,000 cells.

Supplementary Note 3: Data preprocessing.

- **Perturb-Seq data**: The three single-cell RNA-seq datasets used for this study all underwent the same preprocessing. First, each cell was normalized by total counts over all genes and then a log transformation was applied. To reduce the complexity of the prediction problem, we restricted the dataset to only the 5000 most highly varying genes. This is similar to the pre-processing performed by [5] which enabled a more accurate performance comparison (Fig. 2). Since our model requires a gene embedding for every perturbed gene as well, we additionally included any perturbed gene to our dataset that wasn't already accounted for in the set of most highly varying genes. For the analysis of genetic interactions (Fig. 4), we used the gene set from [6] to ensure consistency in our model's predictions as compared to the original analysis on the experimental data in [6]. This was generated by identifying all genes in the raw data that had mean UMI value greater than 0.5.

- **Cell fitness data**: We followed the same procedure as [6]. Genes with a gene growth phenotype of absolute value greater than or equal to 0.05 in a CRISPRa v1 growth screen in K562 were used. The authors compute a genetic interaction (GI) score using these cell fitness measurements. The first step in this procedure is to derive an expectation for the fitness changes that result from combining two genetic perturbations. For each gene fit a quadratic curve is fit to all pairwise fitness measurements involving that gene. GI scores are then defined as the deviation from this expectation. For further details, please read the supplementary information in [6].

Supplementary Note 4: Selection of strong perturbations.

This filtering procedure was only performed on data from the two screens where essential genes were perturbed in [3]. Unfiltered data from these screens is labelled using the notation (E) and filtered data is labelled as (ES). The filtering of perturbations took place at two levels. The first is at the level of perturbations and the second is at the level of individual cells.

- **At the level of perturbations**, we followed the same criteria as used in [3]. Strong perturbations were identified as those that satisfied the following conditions: (i) at least 50 differentially expressed genes at a significance of $p < 0.05$ by Anderson-Darling test following Benjamini-Hochberg correction; (ii) at least 25 cells that passed our quality filters; and (iii) an on-target knockdown, if measured, of at least 30%. This reduced the number of perturbations in the K562 cell line from 2058 to 1092 and in RPE-1 from 2394 to 1543. The number of cells was reduced from 310385 to 192648 for K562 and from 247914 to 175398 for RPE-1.

- **At the level of individual cells**, we tested whether each perturbation was achieving its intended effect by comparing the gene expression level of the perturbed gene (say X) following perturbation to its original level before perturbation. We set as a threshold the expression level of gene X in the tenth percentile cell in the unperturbed control distribution. Any cell where X is perturbed but the expression of X is over this threshold was not considered. It is also important to note that the expression of a perturbed gene is not always measured in the dataset, in which case no filtering was performed. As a consequence of these filterings, the number of cells was reduced from 192648 to 162751 for K562 and from 175398 to 162733 for RPE-1.

Supplementary Note 5: Baseline models.

The following baseline models were used for comparing model performance:

1. **No perturbation model**: This model simply predicts that there was no effect of performing a perturbation and that the unperturbed cell state is the same as the post-perturbed one.

2. **Gene Regulatory Network (GRN) Baseline**: Our GRN-baseline consists of the following steps:

(a) **Gene regulatory network inference**: The first step is to infer the causal network. We used GRNBoost [7] to infer a GRN for each training data split using random forest regression. We retained the top 50 most important edges were retained for each target gene (Supplementary Table 5)

(b) **Learning network edge weights**: The next step is to parametrize the edges of the network For this step, we follow the same approach as that in CellOracle [8]. We use a linear model to predict the expression of each target gene g_i using the expression of all its parents $g_j \in Pa(g_i)$ where $Pa(g_i)$ refers to the parents of g_i in the GRN.

$$g_i = \sum_{g_j \in Pa(g_i)} \beta_i g_j \tag{1}$$

(c) **Simulation of perturbation effect**: Following this, the effect of any applied perturbation was simply linearly propagated along the edges of this graph. Let E represent the weighted adjacency matrix of the learnt graph from the previous step. Let θ represent a genetic perturbation vector, an n-dimensional vector where n is the number of genes. It has a value of zeros at every position except at the indices of genes where perturbations are being applied, where it is either $+1$ or -1. Let $d = 3$ be the number of hops or propagation steps to take along the graph. Then, the perturbation outcomes or change in gene expression $\mathbf{x}_\theta$ caused by a perturbation θ under this model would be:

$$\mathbf{x}_\theta = \left(\prod_{h=1}^{d} \mathbf{E} \right) \cdot \theta \tag{2}$$

3. **Compositional Perturbation Autoencoder (CPA)** [5]: This model uses an adversarial autoencoder with no other prior information to predict the effect of applying a specific perturbation to a given unperturbed cell. We experimented with a few different approaches for incorporating prior knowledge into CPA but didn't observe a substantial improvement in performance (Supplementary Table 6).

   (a) **Average over neighbor embeddings**: We first replaced the initialized embedding of the unseen gene by the average of embeddings of nearby genes on the knowledge graph.

However, this led to no change over the original CPA. We hypothesize that this could be because of two reasons: (i) many genes in the knowledge graph did not have enough seen perturbations as neighbors for the averaging operation to be meaningful (ii) CPA had not performed an aggregation operation over the embeddings of its neighbors at the time of training which would cause the operation to not be very meaningful at the time of inference

(b) **Train an MLP over neighbor embeddings (CPA+KG)**: We also devised a new approach to leverage the KG in the training procedure itself. Given a perturbation, we generated an additional multi-hot feature vector that corresponds to the one-hop neighbor of the query perturbation in the same knowledge graph as used in GEARS. This vector is then encoded with a multi-layer perceptron and the output is summed to the original perturbation one-hot vector during the training stage. When encountering an unseen perturbation, it is learned to use the one-hop neighbor embedding in making the prediction, in a similar spirit as GEARS. This resulted in improved performance over naive CPA as seen in Supplementary Table 6, but has significantly lower performance than GEARS, highlighting the usefulness of many other innovations in GEARS.

Supplementary Note 6: GRN-inference methods and their applicability to multi-gene perturbation outcome prediction.

To the best of our knowledge, there are no published models that can be directly applied to the task of multi-gene perturbation outcome prediction for any gene. Some methods have explored this problem using dynamic or time-varying data [9] but have not done so using data from a single timepoint. We describe below two publications that describe some key approaches that could be applied to this task. In the next section we describe how we used this information to design our own GRN-baseline model.

- **Friedman et al.** [10] introduced the approach of inferring gene regulatory networks or causal networks from gene expression data. They attempt to learn the underlying joint probability distribution between all genes which can be represented as:

$$p_\phi(\mathbf{g}) = \prod_{i=1}^{n} p_\phi(g_i|Pa(g_i)) \tag{3}$$

Here g_i represents any given gene and $Pa(g_i)$ represents the parents of that gene in the causal graph. The joint probability is just the product of the probability of each gene conditioned on its parents. The advantage of using Bayesian networks is that this distribution can be simplified by identifying dependence and conditional independence relations between these genes.

This Bayesian network formulation is general enough to be extended to settings that can account for non-linear perturbation effects. However, the goal in Friedman et al. was only to show that this structure could be learnt as a form of gene expression data analysis. They do not apply this learnt structure to the problem of inferring the effect of a new perturbations, even though this is highlighted as an important direction for future work.

- **Kamimoto et al.** [8], on the other hand, leverage this graph inference formulation to present a perturbation effect modeling framework. This consists of two steps, the first is to learn the underlying gene regulatory network using linear-regression, represented as:

$$y = \sum_i \beta_i x_i + \alpha \tag{4}$$

Here y represents the predicted expression value of a gene and x_i is the expression of its parents i. β_i and α are learnt linear coefficients. The effects of a new perturbation can then simply be predicted by propagating the effects of the applied perturbation down the network. However, even if two disinct nodes in this graph were perturbed and the effects were cascaded down the graph, there is no mechanism for genes to respond non-additively to applied perturbations. Since β_i has no dependence on x_i, there is no second-order term that can predict a non-linear interaction effect. The composition of multiple linear functions is also linear.

- **Other challenges with graph inference** Besides the challenge of predicting non-linear effects, a bigger hurdle within graph inference for gene regulatory networks as a field is the lack of reliable large-scale ground-truth networks. This hinders both the evaluation and development of meaningful improvements to existing algorithms. Most approaches rely on protein-protein interactions or other approximations of the underlying graph which can be

incomplete and severely biased towards more well studied systems. By circumventing network inference entirely, the performance of GEARS can be evaluated and improved more effectively by directly using ground truth perturbation outcome data.

Supplementary Note 7: Designing a gene regulatory network (GRN) baseline and leveraging existing models.

There is significant past work tackling the problem of gene regulatory network (GRN) inference (e.g. using SCENIC [7]), however a GRN does not by itself enable prediction of post-perturbation effects. CellOracle [8], was the first model to attempt to predict perturbation effects using a GRN by learning weights over its edges. However, CellOracle cannot predict outcomes for perturbing any gene and is only restricted to the perturbation of transcription factors. Thus, for our GRN baseline we designed a new model (GRN-baseline) that leverages elements of SCENIC [7] for network inference and elements of CellOracle [8] for perturbation outcome prediction, as described below:

- **SCENIC** has been validated across multiple studies and benchmarks for its ability to infer gene regulatory networks [11]. It is composed of three steps: gene regulatory network inference (using a package called GRNBoost), transcription-factor (TF) motif based filtering of graph edges (using a package called RCISTarget) and TF-regulatory module activity scoring (AUCell). We only make use of the first step for gene regulatory network inference over all genes in the dataset. We disregard the second step since we are not restricting our model to only make predictions for transcription factor perturbation. We disregard the third step since knowledge of regulatory module activity between cells is not relevant to our prediction task.

- **CellOracle** learns linear weights over edges in a gene regulatory network to computationally simulate the effect of gene perturbation. While CellOracle also infers a gene regulatory network, we chose not to use their implementation for network inference because:

  (a) CellOracle requires a starting gene regulatory network that is inferred from scATAC-seq data. Thus, it is limited only to the analysis of TF perturbations.

  (b) The network inference procedure used in SCENIC (GENIE3) has been validated through numerous additional studies, including a formal benchmark [11]. Even on the evalua-

tion within the CellOracle paper itself, SCENIC showed very comparable performance with CellOracle both with and without the base gene regulatory network [8].

Following the network inference step, CellOracle learns linear weights for each edge connecting a TF with one of its downstream genes. Using this weighted network, CellOracle predicts the change in expression following genetic perturbation and models this as a shift in differentiation trajectories for each cell. Our baseline also learns linear weights over the edges similar to CellOracle but predicts perturbation outcome directly. In light of the changes needed to the CellOracle model in order to apply it to the full set of perturbations that GEARS was applied to, it was more efficient for us to implement our own version (GRN-baseline).

- **GRN model evaluation**: For a comprehensive evaluation, we directly compared the performance of GEARS with that of the GRN-baseline as well as CellOracle on the subset of genes where all models can be applied directly (i.e. transcription factor perturbations). For the Norman et al. [6] dataset, this accounted for 102 out of a total of 284 perturbations in the dataset. We observe that GEARS significantly outperforms both models (Supplementary Figure 20) while GRN-baseline outperforms CellOracle. This result highlights why we had to build our own GRN-baseline since no existing GRN-based method is designed to directly predict absolute post-perturbation gene expression.

  To train CellOracle, we used the entire Norman et al. 2019 dataset. We performed perturbations using the $simulate\_shift()$ function as described in the CellOracle online tutorial [12]. The evaluation can be reproduced using code in our repository.

Supplementary Note 8: Identification of most significant differentially expressed genes.

For computing the mean sqaured error over the top-20 most differentially expressed genes, genes were selected based on those that showed the highest absolute differential expression upon perturbation. The unit of measuring gene expression was log normalized counts. In the single-cell analysis literature, a common procedure is to first normalize the counts within each cell such that they sum to a specific value (usually set to be the median sum across all cells in the dataset). Then the values are log transformed (using the natural logarithm).

Both for normalization and ranking genes by differential expression, we used the Scanpy software [13]. We use the function $sc.tl.rank\_genes\_groups()$ with default paramaters including a t-test for estimating scores (API Documentation (Accessed November 2022) [13]). This function will return a z-score for each gene and rank genes by absolute values of the score. We don't include genes for this metric that show a significant level of dropout.

Supplementary Note 9: Identification of known clusters of post-perturbation transcriptional effect within previously un- seen perturbations.

5 different training and testing splits of the Replogle et al. (2022) [3] K562 (ES) dataset consisting of 1093 perturbations were generated. Each split consists of 821 training perturbation and 272 testing perturbations, with minimal overlap between splits (all tests sets combined account for 827 unique perturbations). Within each test set, we identified clusters of transcriptional activity using the true experimental Perturb-Seq data (Extended Data Figure 1a) and then tested GEARS ability to predict these clusters (Extended Data Figure 1b). All clustering was performed using Leiden clustering set to a constant resolution of 0.2 which appeared to capture most visibly distinct clusters.

Note that GEARS had never seen any of the genes in the test set experimentally perturbed at the time of training. We see that GEARS detects these distinct phenotypic clusters significantly better than the model that only predicts mean perturbation effect or no perturbation effect (Extended Data Figure 1c,d). This highlights that GEARS is able to learn meaningful biological effects that wouldn't be possible if it was only learning background correlation.

Supplementary Note 10: Generating a data split for model evaluation.

To generate a data split for the Norman et al. dataset which contained both single and 2-gene perturbations [6], we first randomly sample $K_G\%$ from the gene list and consider them as the gene set that is seen at the time of training. Thus, all single-gene perturbations with genes belonging to this set are used for training. The rest of the genes (1-K_G)% are used as the unseen gene set and the corresponding single-gene perturbations are used for testing. Next, within the 2-gene combination perturbations, in the case when both individual perturbations are in the seen set (0 unseen of 2), we randomly sample $K_C\%$ of them as training perturbations and the rest (1-K_C)% are held out in the test set. For the other 2 categories: 1/2 unseen and 2/2 unseen, we simply

hold out all 2-gene combinations where at least one of the individual genes being perturbed in that combination is in the unseen set. See Extended Figure 1 for an illustration. In our study, we set $K_G = 75, K_C = 75$ to obtain the train+validation and test set and then in the train+valid set, we run $K_G = 90, K_C = 90$ to obtain the train and validation set. In the case of datasets containting only single-gene perturbations, we only test performance on single-gene perturbations which were not seen perturbed at the time of training (1 unseen of 1).

Supplementary Note 11: Comparison of differentially expressed genes.

Statistically significant differential expressed genes with an absolute value of log-fold change greater than 1 ($q < 0.05$) were identified for both the true post-perturbation distribution and the predicted post-perturbation distribution. We used the Mann-Whitney rank test (Wilcoxon rank-sum test) from the diffxpy package for this analysis [14]. We considered all genes with mean log-normalized counts greater than 0.01, as measured on the full dataset. This resulted in 1400 genes from [6] being considered in the analysis out of the original 5045 genes that were used to train the model.

For each perturbation, we measured the overlap between the true set of differentially expressed genes and the predicted set of differentially expressed genes. We used a hypergeometric distribution to measure the statistical significance of this overlap (Extended Data Figure 4). Here the parameters of the hypergeometric distribution were set as: N =Total number of genes, K =Number of true differentially expressed genes, n =Number of predicted differentially expressed genes, k =overlap between predicted and true set of differentially expressed genes. The probability mass function for this distribution is then calculated as:

$$p_X(k) = \Pr(X = k) = \frac{\binom{K}{k}\binom{N-K}{n-k}}{\binom{N}{n}} \tag{5}$$

We see that across all perturbations the set of gene predicted by GEARS to be differentially expressed genes are significantly enriched for true differentially expressed genes (Extended Data Figure 4b).

Supplementary Note 12: Model ablation analysis across generalization conditions.

The effect of the knowledge graph is most visible when making predictions for perturbing genes that have not been seen perturbed at all at the time of training. To illustrate this further, we

designed a model ablation analysis under a wide set of generalization scenarios (1/2 genes seen at the time of training, 2/2 genes seen etc.) (Extended Data Figure 5). Below, we characterize the influence of some key model components based on these results:

- **Knowledge graphs**: In the case where the prediction is being made for at least one gene that has not been seen perturbed previously (1/1 Unseen, 2/2 Unseen and 1/2 Unseen), the addition of the background knowledge graph improves performance much more significantly than in the case where both genes have been seen perturbed previously. The error bars are wider in the case of 0/1 Unseen because the number of perturbations with just a single unseen perturbation in each test split is much smaller than the number of combinations that contain these unseen perturbations.

- **Cross-gene layer**: In the case where genes have been seen perturbed previously, the key prediction being made is for the detection of non-linear interactions between perturbation effects. For instance, in the case of predicting the perturbation outcome for 0/2 genes unseen, the non-trivial prediction being made is for the non-additive interaction between these two genes. This is enabled through (a) the cross-gene layer which enables better generalization of secondary perturbation effects across genes following initial perturbation and (b) multi-dimensional gene embedding which gives the model sufficient room to capture heterogeneous perturbation effects.

- **MSE Loss**: Another key trend visible here is that the autofocus loss has a significant impact on improving performance across all settings.

Supplementary Note 13: Generating UMAPs and clustering of post-perturbation gene expression data.

The UMAPs in Figure 4b and Figure 4c were generated using GEARS-predicted post-perturbation gene expression profiles of all pairwise combinations of 102 1-gene perturbations from the the Norman et al. [6] dataset. Out of a total of 105 single gene perturbed in the dataset, for this figure, we used data for the perturbation of 102 genes that were present in the Gene Ontology database for Homo sapiens species. Figure 4c shows this complete UMAP with post-perturbation

outcomes for all possible 5151 2-gene perturbations as well as the 102 1-gene perturbations. Figure 4b was plotted using GEARS-predicted post-perturbation gene expression profiles of only those perturbations from [6] (102 1-gene perturbations and 128 2-gene perturbations) used to train GEARS. Thus, Figure 4b is a subset of the data in Figure 4c. The UMAP manifold was computed only once for both figures to enable a direct comparison.

Clustering was performed using Leiden clustering with default parameters set in scanpy (resolution = 1). Clusters shown in Figure 4b and 4c were labelled using phenotypic labels from Norman et al. [6]. If any cluster or set of clusters contained perturbations that were labelled as exhibiting a specific phenotype in Norman et al., then that whole cluster or set of clusters was labelled as showing that specific phenotype in Figure 4b or 4c4. All other clusters were not given phenotypic labels in these figures.

Supplementary Note 14: Differential expression analysis for novel phenotypic cluster identified by GEARS.

The novel phenotypic cluster identified by GEARS showed high expression of known erythroid lineage-specific marker genes. *HBG1, HGB2, HBZ, HGA1, HBA2, GYPA, ERMAP* were identified as erythroid lineage-specific marker genes in [6]. Out of these, *HBG1, HBZ, HBA2, GYPA* were present among the genes whose expression GEARS was trained to predict (Methods). We see a higher expression of all these four marker genes in the new phenotypic cluster than in any experimentally tested perturbation (Supplementary Figure 10)

For further validation of the novel phenotypic cluster discovered by GEARS we used single-cell gene expression data from Tabula Sapiens. We chose to use the data from the bone marrow organ as it contains hematopoetic progenitor cells, which is the cell type most similar to the K562 lymphoblasts that were used for perturbational experiments in Norman et al. [6]. The bone marrow dataset also contains all the cell types that hematopoetic progenitor cells can differentiate into. We identified proerythroblasts as a cell type that represents an early stage in the erythroid lineage. The differential expression (DE) between hematopoetic progenitor cells and proerythroblasts was use to represent the transition to an early stage in the erythroid lineage in Tabula Sapiens. Differentially expressed genes were identified using scanpy and the log fold change in expression was computed using diffxpy. Any gene with absolute value of log fold change in expression greater than 10 or

less than 0.01 was not considered. A vector $v_e = [\Delta g_1, \Delta g_2 \dots, \Delta g_k]^T$ consisting of the log fold change in expression for all k differentially expressed genes was constructed for the transition from hematopoetic progenitor cells to proerythroblasts. A similar DE vector v_p for the same k genes was constructed for each of the perturbations that GEARS predicted the outcome for. A dot product $v_e \cdot v_p$ was computed between the DE vector for each perturbation and the DE vector for the transition to proerythroblasts (Supplementary Figure 10). We see that the new phenotypic cluster exhibits a novel phenotype that is most similar to the transition to proerythroblasts in Tabula Sapiens. While this indicates that the phenotype observed is biologically meaningful, it has not yet been experimentally validated. We further confirmed the robustness of this prediction by removing all perturbations that produce a similar phenotype from the training set (Supplementary Figure 11).

Supplementary Note 15: Measuring genetic interaction scores.

For identifying and categorizing genetic interactions we followed the definitions and metrics defined in Norman et al. [6]. They defined the following types of GIs: additive, epistatic, neomorphic, potentiation, redundant, suppressive, synergy (similar/dissimilar). The authors make a distinction between synergistic combinations based on the similarity of the combining single-gene perturbations. We did not include this division because our focus was on evaluating predictions for combinatorial perturbations. We also did not include 'potentiation' as a separate category and instead grouped it under synergy. This is because it was defined as the combined interaction of high synergy and epistasis and we evaluated those GIs individually. 'Additive' interactions (or the no-GI class), which are defined as the complement of seeing either synergy or suppression are only included in Extended Data Figures 4, 5.

Norman et al. [6] defined metrics (GI scores) for identifying GIs using a linear model of the combinatorial perturbation effect. Let $\mathbf{g}^i \in \mathbb{R}^K$ be the post-perturbation gene expression vector of a cell i with K genes. Let $\mathcal{C}_k$ be the set of cells under perturbation k, where $|C_k| = T_k$. The first step is to compute the average post-perturbation gene expression ($\bar{\mathbf{g}}^k$) for each of the two combining genes a, b perturbed singly as well as in combination $(a + b)$:

$$\bar{\mathbf{g}}^k = \frac{1}{T_k} \sum_{i \in \mathcal{C}_k} \mathbf{g}^i, \qquad \text{where } k \in \{a, b, (a+b)\}$$

	Metric (GI score)	Definition	Relevant GI
1	Magnitude	$\sqrt{c_a^2 + c_b^2}$	Synergy, Suppression, Additivity
2	Similarity of (single/double) transcriptional profiles	$corr([\mathbf{a}, \mathbf{b}], \mathbf{ab})$	Redundancy
3	Model fit	$corr(c_a\mathbf{a} + c_b\mathbf{b}, \mathbf{ab})$	Neomorphism
4	Equality of contribution	$\frac{\min(dcor(\mathbf{a},\mathbf{ab}),dcor(\mathbf{b},\mathbf{ab}))}{\max(dcor(\mathbf{a},\mathbf{ab}),dcor(\mathbf{b},\mathbf{ab})))}$	Epistasis

Table 1: Metrics used to define genetic interaction sub-types from gene expression data.

Then the change over mean expression in unperturbed control cells ($\bar{\mathbf{g}}^{ctrl}$) is computed as:

$$\delta\bar{\mathbf{g}}^k = \bar{\mathbf{g}}^k - \bar{\mathbf{g}}^{ctrl}$$

And it is used to fit the following linear model:

$$\delta\bar{\mathbf{g}}^{(a+b)} = c_a\delta\bar{\mathbf{g}}^a + c_b\delta\bar{\mathbf{g}}^b + \epsilon \tag{6}$$

Here ϵ captures the error in the model fit. Following the procedure in Norman et al [6], the model was fit using robust regression with a Theil-Sen estimator (fit on 10,000 random subsamples of 1,000 genes at a time) Using the values of the coefficients, the following metrics (or GI scores) were defined shown below. To simplify the notation we write $\delta\bar{\mathbf{g}}^{(a+b)}$ as **ab**, $\delta\bar{\mathbf{g}}^a$ as **a** and $\delta\bar{\mathbf{g}}^b$ as **b**.

Here, $corr$ refers to a distance correlation and the square brackets represent the concatenation operation. When predicting a GI score, first the mean post perturbation expression vectors are predicted for both the combination perturbation and the single-gene perturbations ($\delta\hat{\bar{\mathbf{g}}}^{(a+b)}, \delta\hat{\bar{\mathbf{g}}}^a, \delta\hat{\bar{\mathbf{g}}}^b$). These are then used to estimate the relevant parameters such as in (6). When calculating the true value for the GI score, the same procedure is performed with true post perturbation gene expression vectors ($\delta\bar{\mathbf{g}}^{(a+b)}, \delta\bar{\mathbf{g}}^a, \delta\bar{\mathbf{g}}^b$).

Supplementary Note 16: Identifying genetic interaction subtypes.

For each defined GI subtype q, the authors in [6] defined a set of 2-gene combinatorial perturbations $\mathbf{S}_q$ as expressing that type of interaction. However, they did not explicitly state the GI score thresholds used to define these sets. To estimate these thresholds, we first computed the relevant GI score for every element belonging to a given GI subtype set $\mathbf{S}_q$ using true post-perturbation gene expression. We then estimated the minimum score in case of a lower bounded

Genetic Interaction (GI)	Defintion
Synergy	Magnitude > 1.15
Suppressive	Magnitude < 1.0
Neomorphism	Model fit < 0.88
Redunant	Similarity of (single/double) transcriptional profiles > 0.85
Epistasis	Equality of Contribution > 0.28

Table 2: Thresholds used to define genetic interaction sub-types.

condition and the maximum score in case of an upper bounded condition and used this as the score threshold τ_q for each GI subtype q. These thresholds are also visualized as colored horizontal lines in Extended Data Fig 5. The result was the following conditions for labeling an interaction as belonging to a specific GI subtype. Overall, no GI subtype set accounted for more than 50% of all 131 combinations being tested.

Supplementary Note 17: Model evaluation for predicting genetic interactions.

We evaluated GEARS's ability to correctly predict different GI subtypes. A leave-one-out testing procedure was followed for this analysis (Figure 3, Extended Data Figure 5, 6, 7, 8). For every combinatorial perturbation experimentally tested in Norman et al. [6], we trained GEARS from scratch while only holding out that specific interaction in the test set. Thus, we trained 131 different models. We performed the same procedure with the deep learning-based baseline model CPA [5].

Once each model was trained, we computed all the GI scores (Table 1) for the perturbation that was held out in the test set. Using thresholds from Table 2, we identified whether a specific GI was predicted to exhibit a specific GI subtype. The same procedure was also performed using true post perturbation gene expression.

The performance of GEARS in predicting each GI subtype was evaluated using the following metrics

- **Precision**: The fraction of combinatorial perturbations predicted to show a specific GI subtype that were also identified to do so based on true post-perturbation expression. Let $\hat{\mathbf{S}}_q$ be the set of perturbations predicted to show a specific GI subtype and $\mathbf{S}_q$ be the perturbations

that truly show that GI subtype.

$$\text{Precision} = \frac{|\hat{\mathbf{S}}_q \bigcap \mathbf{S}_q|}{|\hat{\mathbf{S}}_q|}$$

- **Recall**: The fraction of combinatorial perturbations that were identified as showing a specific GI subtype based on true post perturbation gene expression that were also predicted to do so by the model being evaluated.

$$\text{Recall} = \frac{|\hat{\mathbf{S}}_q \bigcap \mathbf{S}_q|}{|\mathbf{S}_q|}$$

- **Accuracy**: The fraction of 2-gene perturbations that were correctly identified as either showing or not showing a genetic interaction as compared to classification based on true post perturbation gene expression. Let $\hat{\mathbf{S}}_q^c$ be the set of perturbations predicted to not show a specific GI subtype and $\mathbf{S}_q^c$ be the perturbations that truly don't show that GI subtype.

$$\text{Accuracy} = \frac{|\hat{\mathbf{S}}_q \bigcap \mathbf{S}_q| + |\hat{\mathbf{S}}_q^c \bigcap \mathbf{S}_q^c|}{|\mathbf{S}_q| + |\mathbf{S}_q^c|}$$

- **Precision@10**: Of the 10 combinatorial perturbations predicted to have the highest GI score for a given GI subtype, precision@10 refers to the fraction that were truly identified as belonging to that GI subtype using true post-perturbation expression. For example, the ten combinatorial perturbations with the highest score for magnitude were used to evaluate precision@10 for synergistic interactions while those with the lowest were used to do the same for suppressive interactions. Let $\hat{\mathbf{S}}_q^{10}$ be the set of 10 combinatorial perturbations predicted by a model to have the highest GI score for a given GI subtype. Here $|\mathbf{S}_q| \geq 10$.

$$\text{Precision@10} = \frac{|\hat{\mathbf{S}}_q^{10} \bigcap \mathbf{S}_q|}{|\hat{\mathbf{S}}_q^{10}|}$$

In practice, it is more common for scientists to choose a handful of promising combinations to test experimentally as opposed to exhaustively testing all likely combinations. Thus, by focussing on the model's ability to correctly rank the most likely genetic interactions, precision@10 captures the success probability of follow-on experiments that aim to validate model predictions. We compared our performance to a random baseline by drawing 1000 random sets of 10 combinations from this set and plotting their mean and standard deviation

as a null model. This set of 131 combination perturbations was slightly biased towards the presence of an interaction, thus the random baseline helps to put our predictive performance in context. We did not use the naïve baseline that assumed that the combination perturbation effect would be a simple sum of the single-gene perturbation effects, because this would trivially result in the same GI score for all combinations.

- **Top-10 Accuracy**: Of the 10 combinatorial perturbations predicted to have the highest GI score for a given GI subtype, top 10 Accuracy refers to the fraction that were also identified as being part of the 10 combinatorial perturbations identified to have the highest GI score using true post-perturbation expression. Thus, top-10 accuracy is more robust to biases in the dataset towards oversampling genetic interactions but it is also a more conservative metric for measuring performance. Let $\mathbf{S}_q^{10}$ be the set of 10 combinatorial perturbations identified to have the highest GI score for a given GI subtype as measured using true post-perturbation gene expression.

$$\text{Top-10 Accuracy}= \frac{|\hat{\mathbf{S}}_q^{10} \bigcap \mathbf{S}_q^{10}|}{|\mathbf{S}_q^{10}|}$$

Supplementary Note 18: Model evaluation for predicting non-additive effects.

The GI scores defined above [6] consider the expression values of all genes when calculating the score. Often, it is only the expression of a few genes that manifests an interaction phenotype or a non-additive effect. To focus our analysis on these interacting genes, we measured how many genes post-perturbation were expressed in a manner that was very different from a simple additive effect. We first defined a naïve additive model that simply added together the effects of the individual single gene perturbations. As defined previously, if $\delta\bar{\mathbf{g}}^{(x)}$ represents the mean change in expression over unperturbed control when perturbing gene x, then the naive additive model predicts that the effect of perturbing the combination of genes $(a + b)$ would result in the following effect:

$$\delta\bar{\hat{\mathbf{g}}}_{\text{nv}}^{(a+b)} = \delta\bar{\mathbf{g}}^{a} + \delta\bar{\mathbf{g}}^{b}$$

We used this naive sum to sort genes by how far their true post-perturbation expression under

a combination perturbation deviated from this naïve prediction (Fig. 3a).

$$\text{Deviation} = |\delta\bar{\mathbf{g}}^{(a+b)} - \delta\bar{\hat{\mathbf{g}}}_{\text{nv}}^{(a+b)}|$$

We then measured the mean squared error in predicting the top 20 with the highest deviation across all combination perturbations. The final results were categorized by GI type (Extended Data Figure 6a).

Supplementary Note 19: Validation of genetic interaction map using combinatorial cell fitness screen.

Additional computational analysis was performed to increase our confidence in the prediction of unexpected/strong genetic interactions. We compared GEARS predictions for all 4186 pairwise combinations of 92 single genes considered in the GI map (Figure 5) to results from a combinatorial cell fitness screen run on the same perturbations in [6]. Neither was GEARS trained on cell fitness prediction nor was any Perturb-seq data available to train GEARS for the combinatorial perturbations used for this evaluation. While GEARS predictions measure a much broader and complex phenotype than what is captured in cell fitness, we expected that the strongest cell fitness-based interactions (measured using a cell fitness-based GI score [15], Supplementary Information) would also be detected in the full gene expression profile predicted by GEARS. Thus, we chose the strongest interactions as measured using the cell fitness screen (more than two standard deviations away from the mean cell fitness-based GI score) and compared them to GEARS predictions for those same perturbations.

We see that GEARS predicted GI score for synergy and suppression effectively captures the strong interaction effects detected using the experimental measurements from the cell fitness screen (Extended Data Figure 7). The distribution of GEARS-predicted GI scores is significantly higher ($p < 0.0013, n = 123$, one-sided t-test comparing the means of two independent samples) for perturbations showing strongly synergistic (synthetic lethal) cell fitness effects as compared to those showing approximately additive cell fitness effects ($n = 3141$). Similarly, the distribution of GEARS-predicted GI scores is significantly lower ($p < 4 \times 10^{-5}, n = 69$) for perturbations showing strongly suppressive (buffering) cell fitness effects. We find comparable performance when using post-perturbation gene expression from real experimental Perturb-seq data (Synergy: $p < 2 \times 10^{-6}, n = 22$. Suppression: $p < 6 \times 10^{-5}, n = 11$). 69% of 123 strongly synergistic

combinatorial perturbations (as measured by cell fitness) showed a more synergistic rather than suppressive GI score based on GEARS predictions. This compares favorably to 68% of the 22 perturbations that showed the same trend with real experimental data from a Perturb-Seq assay. In the case of strongly suppressive cell fitness effects, 69% of 69 combinatorial perturbations were identified by GEARS as more suppressive than synergistic while 100% of 11 perturbations did so based on experimental PerturbSeq data. The baseline using real PerturbSeq data is important since the GI score computed using transcriptome-wide information and that computed using cell fitness measure different phenotypes, even if correlated in the case of strong cell fitness phenotypes.

Thus, GEARS provides a meaningful approach for computationally expanding the information gained from Perturb-Seq experiments especially in the case where such data may not be available. Moreover, this also increases our confidence that several strong interactions shown in the GI map predicted by GEARS (Figure 5) are biologically meaningful even if not all predictions have been experimentally validated.

Supplementary Note 20: Predicting endpoint phenotypic effects.

GEARS was trained to directly predict endpoint phenotypic effects along with gene expression by adapting its model architecture (Supplementary Figure 16). The modified model architecture applies a small modification to the output layer that allows for prediction of a desired endpoint phenotypic effect along with gene expression. Supplementary Figure 17 shows the performance of GEARS on predicting the cell fitness phenotype for data from a 2-gene combinatorial cell fitness screen on the K562 cell line [6]. We held out different portions of the training data (40%, 50%, 60%) and measured our performance in predicting the cell fitness values for all the remaining combinations. We see that across all scenarios, GEARS shows very high predictive performance (Coefficient of variation $R^2 = 0.91, 0.90, 0.94$). We also tested our performance on a different dataset, as well as a different cell line (Jurkat), and found very good performance for both ($R^2 = 0.70, 0.89$) (Supplementary Figure 17).

Supplementary Note 21: Analysis of confounding factors.

- **Cell type**: At present, our approach has only been tested on data from the same cell type as that used for training. We currently do not make any claims on transferability of predictions across cell types.

- **Experimental conditions**: Besides cell type, we also recommend that GEARS be used to predict outcomes under the same experimental conditions as those used at the time of training and to limit variation in factors such as the number of UMIs per cell.
- **Cell-cycle effects**: As for cell cycle effects, we recommend that these be minimized between the unperturbed and perturbed distributions through approaches such as subsampling by cell cycle phase. We do not recommend using the commonly-used approach of cell cycle regression since it can adversely influence the gene expression values and affect performance.
- **Success of the gene editing experiment**: GEARS assumes that conventional quality control methods were performed following the perturbation experiment to ensure that the targeted gene was indeed perturbed. We recommend following the guidelines in Replogle et al. 2022 [3] for estimating whether a perturbation was successful or not. GEARS also expects that the vast majority of perturbations/guides were correctly mapped to the correct perturbed cell.
- **Heterogeneity of the post-perturbation distribution**: It is non-trivial to map unperturbed cells to their correct post-perturbed state, since cells are destroyed to measure gene expression. We try to minimize the assumptions we make about how cells should map across these two states by relying on average perturbation effects. This makes a simpler and more general assumption that a significant proportion of the perturbed population is uniformly effected by the perturbation, making the difference in means a good estimator of treatment effect. This is also a reasonable assumption when predicting genetic interaction effects.

Supplementary Note 22: Hyperparameter Search.

We use HyperBand [16] on the validation set of a fixed split of the Norman dataset to find the best hyperparameters. The same set of hyperparameters are then used across all datasets and multiple splits. The set of ranges for the hyperparameters include: GNN architecture – {graph convolutional network (GCN) [17], graph attention network (GAT) [18], simplifying graph convolutional network (SGC) [19]}; GNN layer size – {1, 2, 3}; hidden size d – {32, 64, 128}; autofocus loss coefficient γ – {2, 4}; direction loss regularization term λ – {1, 0.1, 0.01}; the number of top similar genes in the co-expression network H_{pert} – {3, 5, 10, 20}; the number of top similar genes in the perturbation network H_{gene} – {3, 5, 10, 20}; correlation threshold for co-expression network

δ - {0.4, 0.8}; learning rate – {1e-2, 1e-3, 1e-4}; batch size – {32, 64, 128}. Since we have a large set of hyperparameters, for a more efficient selection, we apply HyperBand on different groups of hyperparameters where each group has a small set of hyperparameters while fixing the rest. The final set of hyperparameters are the following: GNN architecture - SGC; GNN layer - 1; hidden size - 64; γ - 2; $\lambda - 0.1$; H_{pert} - 20; H_{gene} - 5; δ = 0.4; learning rate - 1e-3; batch size - 32.

Supplementary Note 23: Selecting predictions with low uncertainty.

GEARS is able to predict an uncertainty value $s_u = \log \sigma_u^2$ for each gene u. To generate a transcriptome-level uncertainty value, we simply took the mean across all model-predicted uncertainty values for all genes. So, for some cell i, we estimated its uncertainty value as the following:

$$\mathbf{s}^i = \frac{1}{K}\sum_{u=1}^{K} s_u$$

To allow comparison of this uncertainty value across different models, we performed z-score normalization using the mean and standard deviations of the predicted uncertainty values for all the data used to train that model. If $\mathcal{C}_{tr}$ are the cells in the training data, we first calculate the mean μ_{tr} and standard deviation σ_{tr} of the set of uncertainty values $\{\mathbf{s}^i : \forall i \in \mathcal{C}_{tr}\}$.

We can then z-score normalize the uncertainty values for any cell j across different trained models as follows:

$$z^j = \frac{s^j - \mu_{tr}}{\sigma_{tr}}$$

Supplementary Tables

Supplementary Table 1: Performance Metrics

Metric	Description	Figure
Mean Squared Error (MSE)	Average squared difference between the predicted and true post-perturbation gene expression. Usually measured for some subset of the most differentially expressed (DE) genes as identified using true post-perturbation gene expression.	Ext. Data Figs. 3, 6, Supplementary Fig. 1, 8
Normalized MSE	MSE value normalized to the MSE measured under a baseline model that predicts no effect for any perturbation.	Fig. 2, Supplementary Figs. 3, 5, 19, 20
Pearson Correlation	Pearson correlation between the predicted absolute post-perturbation gene expression and the true post-perturbation gene expression.	N/A
Pearson Correlation (Delta Expression)	Pearson correlation between the predicted change in post-perturbation gene expression over unperturbed control expression and the true change in post-perturbation gene expression over control.	Fig. 2, Ext. Data Fig. 4, Supplementary Figs. 2, 8, 9, 19, 20
Percentage of top 20 DE genes with opposite direction	The percentage of top 20 most differentially expressed genes for a given perturbation that are predicted to change their expression in response to the perturbation in a direction that is opposite to the true direction of change.	Fig. 2, Supplementary Fig. 8
Jaccard similarity with true differentially expressed genes	Jaccard similarity between the predicted set of DE genes following perturbation and the true set.	Fig. 2

Table 1: Performance Metrics (continued)

Metric	Description	Figure
Precision	The proportion of 2-gene perturbations predicted to show a specific genetic interaction that also showed that interaction based on true post-perturbation gene expression.	Ext. Data Fig. 6
Precision@10	Precision measured only over the set of 10 2-gene combinations that were predicted to show the strongest genetic interaction phenotype.	Fig. 3
Recall	The fraction of 2-gene perturbations that showed a genetic interaction based on true post-perturbation gene expression that were also predicted as such by the model.	Ext. Data Fig. 6
Accuracy	The fraction of 2-gene perturbations that were correctly identified as either showing or not showing a genetic interaction as compared to classification based on true post-perturbation gene expression.	Supplementary Fig. 12
Top-10 Accuracy	The fraction of the 10 2-gene perturbations that showed the strongest interactions based on true post-perturbation gene expression that were also detected as such by the model. This metric compares sets and is invariant to the ranking of perturbations within the top-10 predictions.	Ext. Data Fig. 6
Fraction of Top 20 DE genes with predictions in +/- 25 % of true perturbation variation	The fraction of DE genes where the predicted post-perturbation gene expression value falls within 25%-ile around the mean of the true post-perturbation distribution.	Supplementary Fig. 1, 5
Percentage of top 20 DE genes within 1 SD	The fraction of DE genes where the predicted post-perturbation gene expression value falls within 1 standard deviation around the mean of the true post-perturbation distribution.	Supplementary Fig. 8

Table 1: Performance Metrics (continued)

Metric	Description	Figure
Average of Z-Score across Top 20 DE genes	Average of the z-score of the predicted post-perturbation expression value for each of the top 20 DE genes. Each z-score is computed over the true post-perturbation expression distribution for that gene using its true mean and standard deviation.	Supplementary Fig. 1, 5
Difference of Fold Change between prediction and true expression	Difference in predicted post-perturbation fold change in gene expression and true post-perturbation fold change.	Supplementary Fig. 1, 5
Adjusted Rand Index (ARI)	Rand Index is a similarity measure between two clusterings that considers all pairs of samples and counts pairs that are assigned in the same or different clusters. ARI is Rand Index adjusted for chance.	Extended Data Fig. 1
Normalized Mutual Information	A measure of the mutual dependence between two random variables, used to evaluate clusterings. Here, random variables correspond to the true and predicted cluster assignments of the data. Normalized to be between 0 and 1.	Extended Data Fig. 1

Author	Cell Type	# Pert- -urbations	# Cells	Type	ΔMSE (Top-20 DE)	ΔPearson DE (All Genes)
Jost 2022 [20]	K562	26	23,297	1-gene	-9.1%	+263.1%
Dixit 2016 [21]	K562	20	44,375	1-gene	-64.3%	+12,765.3%
Tian 2019 [22]	Neurons	27	54,095	1-gene	-35.9%	+8,423.9%
Adamson 2016 [23]	K562	87	68,603	1-gene	-52.5%	+349.1%
Tian 2019 [22]	iPSC	27	104,768	1-gene	+11.2%	+8,826.4%
Replogle 2022 (ES) [3]	RPE1	1,543	175,398	1-gene	-48.9%	+382.9%
Replogle 2022 (ES) [3]	K562	1,092	192,648	1-gene	-29.2%	+499.4%
Replogle 2022 (E) [3]	RPE1	2,394	247,914	1-gene	-33.7%	+893.5%
Replogle 2022 (E) [3]	K562	2,058	310,385	1-gene	-10.6%	+547.2%
Replogle 2022 (GW) [3]	K562	9,867	1,989,578	1-gene	+0.25%	+136.3%
Replogle 2020 [24]	K562	87	37,238	2-gene	-17.4%	+36%
Norman 2019 [6]	K562	283	91,205	2-gene	-54.2%	+27.4%
					Lower better	Higher better

Supplementary Table 2: GEARS outperforms baselines across multiple datasets: Performance improvement in predicting post-perturbation gene expression when comparing GEARS with all baselines. MSE is the mean squared error between the post-perturbation gene expression predicted by the model to true post-perturbation gene expression. Pearson DE is the Pearson correlation between the change in gene expression over unperturbed control as predicted by the model to the true change in post-perturbation gene expression. Δ refers to the change in the value of the metric when comparing GEARS to the next best performing baseline. **E**: Essential genes, **ES**: Only strong perturbations within essential genes (Methods), **GW**: Genome-wide perturbation.

Dataset	# perts.	# cells	GRN Baseline	GEARS
Jost 2022	26	23,297	$<$ 2 hours	$\sim$ 23 min
Replogle 2020 - K562	87	37,238	$<$6 hours	$\sim$ 30 min
Tian 2019-Neurons	27	54,095	$<$6 hours	$\sim$ 30 min
Tian 2019-iPSC	27	104,768	$<$1 day	$\sim$ 1 hour
Replogle 2022 (ES) - RPE1	1,543	175,398	$\sim$4 days	$\sim$ 2 hours
Replogle 2022 (ES) - K562	1,092	192,648	$\sim$4 days	$\sim$ 2 hours
Replogle 2022 (E) - RPE1	2,394	247,914	N.A	$\sim$ 4 hours
Replogle 2022 (E) - K562	2,058	310,385	N.A	$\sim$ 4 hours
Replogle 2022 (GW) - K562	9,867	1,989,578	N.A	$\sim$ 1 day

Supplementary Table 3: Scalability of GEARS across different datasets.

PMID	Author	Avg. # of perts. in split	Sub-group	ΔMSE	Δ Pearson DE
35688146	Replogle 2020	4.3	0/2 Genes Seen	+4%	+79%
35688146	Replogle 2020	14.2	1/2 Genes Seen	-18%	+18%
35688146	Replogle 2020	5.4	2/2 Genes Seen	-13%	+5%

Supplementary Table 4: Performance improvement across generalization conditions for a 2-gene perturbation dataset. Δ refers to the change in the value of the metric when comparing GEARS to the next best performing baseline.

Network Inference	Edge Filtering	Weight Learning	MSE (Top20)	Pearson DE
No Perturb	N/A	N/A	0.551 ± 0.029	0.004 ± 0.006
Gene Coexpression	Top-50	Linear	0.524 ± 0.030	0.134 ± 0.007
Gene Coexpression	Top-50	Lasso	0.525 ± 0.029	0.131 ± 0.005
Gene Coexpression	95 percentile	Linear	0.526 ± 0.028	0.133 ± 0.006
Gene Coexpression	95 percentile	Lasso	0.525 ± 0.031	0.129 ± 0.009
SCENIC (GENIE3)	Top-50	Linear	$\mathbf{0.472 \pm 0.033}$	0.188 ± 0.007
SCENIC (GENIE3)	Top-50	Lasso	0.520 ± 0.029	0.084 ± 0.009
SCENIC (GENIE3)	95 percentile	Linear	0.474 ± 0.028	$\mathbf{0.200 \pm 0.005}$
SCENIC (GENIE3)	95 percentile	Lasso	0.521 ± 0.031	0.086 ± 0.005
Gene Ontology	N/A	Linear	0.536 ± 0.055	0.129 ± 0.007

Supplementary Table 5: Performance comparison of different network inference based baselines. Models were trained and evaluated on 5 splits using the Norman et al. 2019 [6] dataset. Top-50 corresponds to the top 50 edges for each target gene as ranked by the importance score. 95 percentile retains all edges with importance score above the 95th percentile of all scores.

Model Name	Changes to Method	MSE	Pearson DE
No Perturb	-	0.551 ± 0.029	0.004 ± 0.006
CPA	Original	0.354 ± 0.049	0.440 ± 0.036
CPA + KG	MLP over KG neighbors	0.333 ± 0.046	0.504 ± 0.029
GEARS	-	$\mathbf{0.216 \pm 0.053}$	$\mathbf{0.556 \pm 0.030}$

Supplementary Table 6: Performance comparison of different modifications to CPA using a knowledge graph (KG). Results shown here for data from Norman et al. 2019 [6].

Supplementary Figures

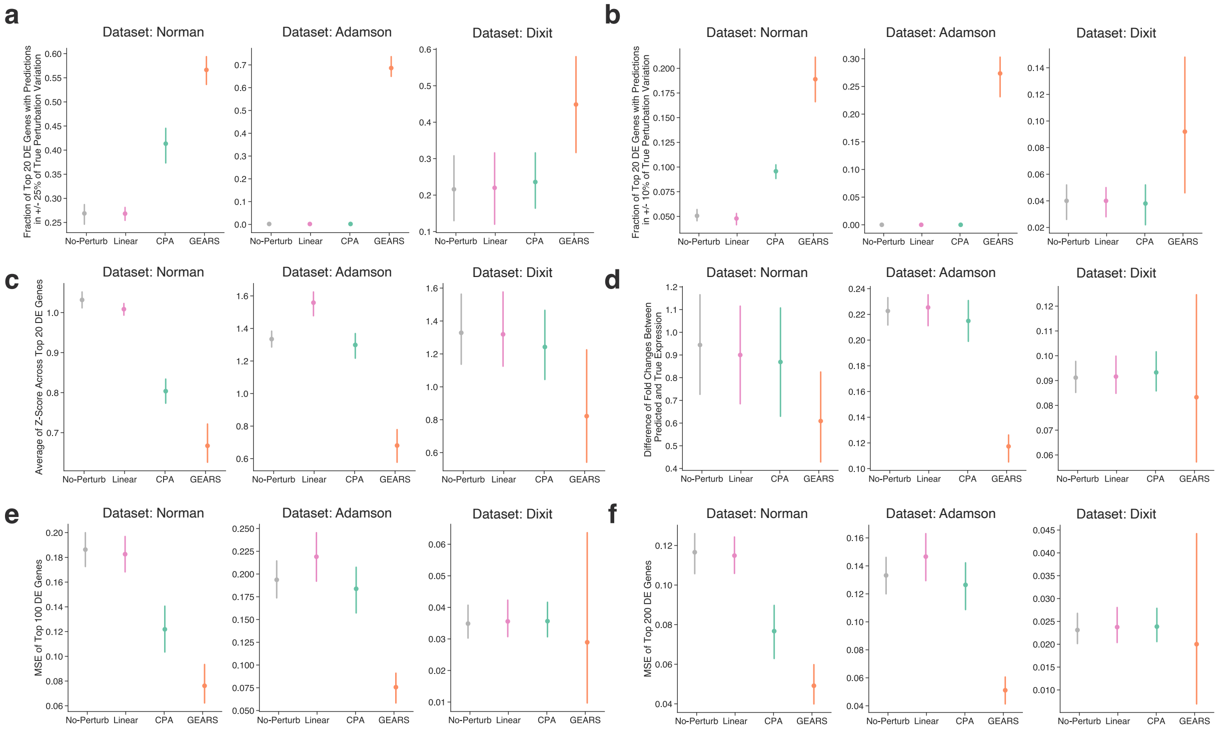

Supplementary Fig. 1: Comprehensive evaluation establishes robustness of GEARS's prediction of post-perturbation expression. **(a)** Fraction of the top 20 differentially expressed genes for each perturbation that have predicted post-perturbation expression within the 40th percentile and the 60th percentile of the true post-perturbation expression. **(b)** Fraction of the 20 most differentially expressed genes for each perturbation that have predicted post-perturbation expression within +/- 25% of true post-perturbation expression variation. This corresponds to the interval between the 25th percentile and the 75th percentile of the true post-perturbation expression. **(c)** Measuring variability in predictions using the average Z-Score across top 20 differentially expressed genes. Z-score was computed using the mean and standard deviation of the true post-perturbation expression distribution for each gene after each perturbation. **(d)** Fold change between predicted post-perturbation expression and true expression. **(e)** MSE in predicted post-perturbation expression for top 100 differentially expressed genes. **(f)** MSE in predicted post-perturbation expression for top 200 differentially expressed genes. For all panels **(a-f)** the marker indicates the mean value of the corresponding metric as measured over predictions made by models trained using 5 different training data splits (n=5). The error bars represent bootstrapped 95% CI.

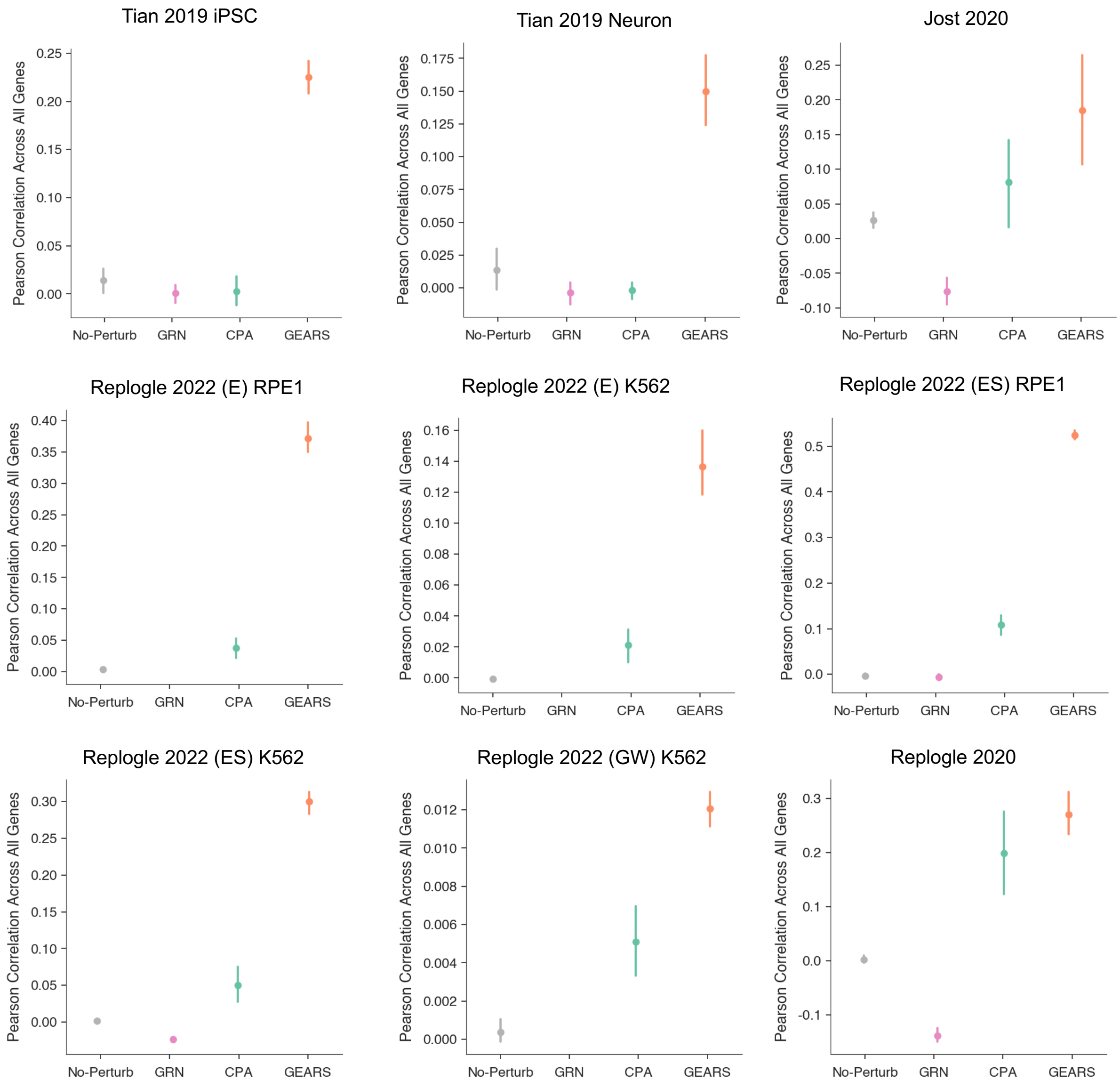

Supplementary Fig. 2: GEARS shows consistent improvement in performance (Pearson correlation) over baselines across 7 datasets with diverse cell types, number of perturbations and experimental conditions. Improvement was measured using the Pearson correlation between the mean model predicted change in gene expression over control (delta expression) to true mean change in gene expression over control. For all panels, the marker indicates the mean Pearson correlation over predictions made by models trained using 5 different training data splits (n=5). The error bars represent bootstrapped 95% CI.

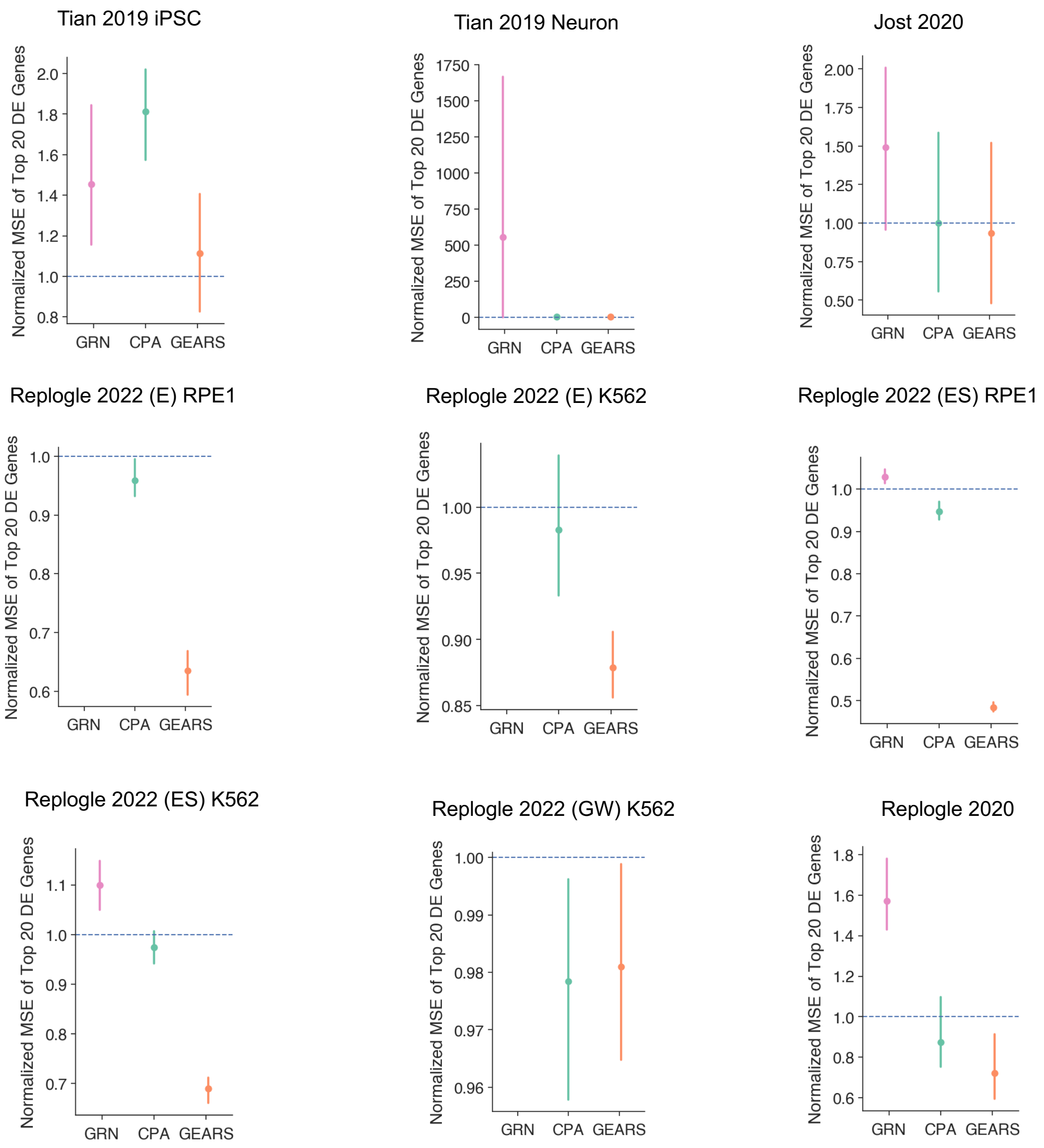

Supplementary Fig. 3: GEARS shows consistent improvement in performance (MSE) over baselines across 7 datasets with diverse cell types, number of perturbations and experimental conditions. Improvement was measured using the normalized mean sqaure error between the model predicted post-perturbation gene expression and true post-perturbation gene expression. For all panels, the marker indicates the mean MSE over predictions made by models trained using 5 different training data splits (n=5). The error bars represent bootstrapped 95% CI.

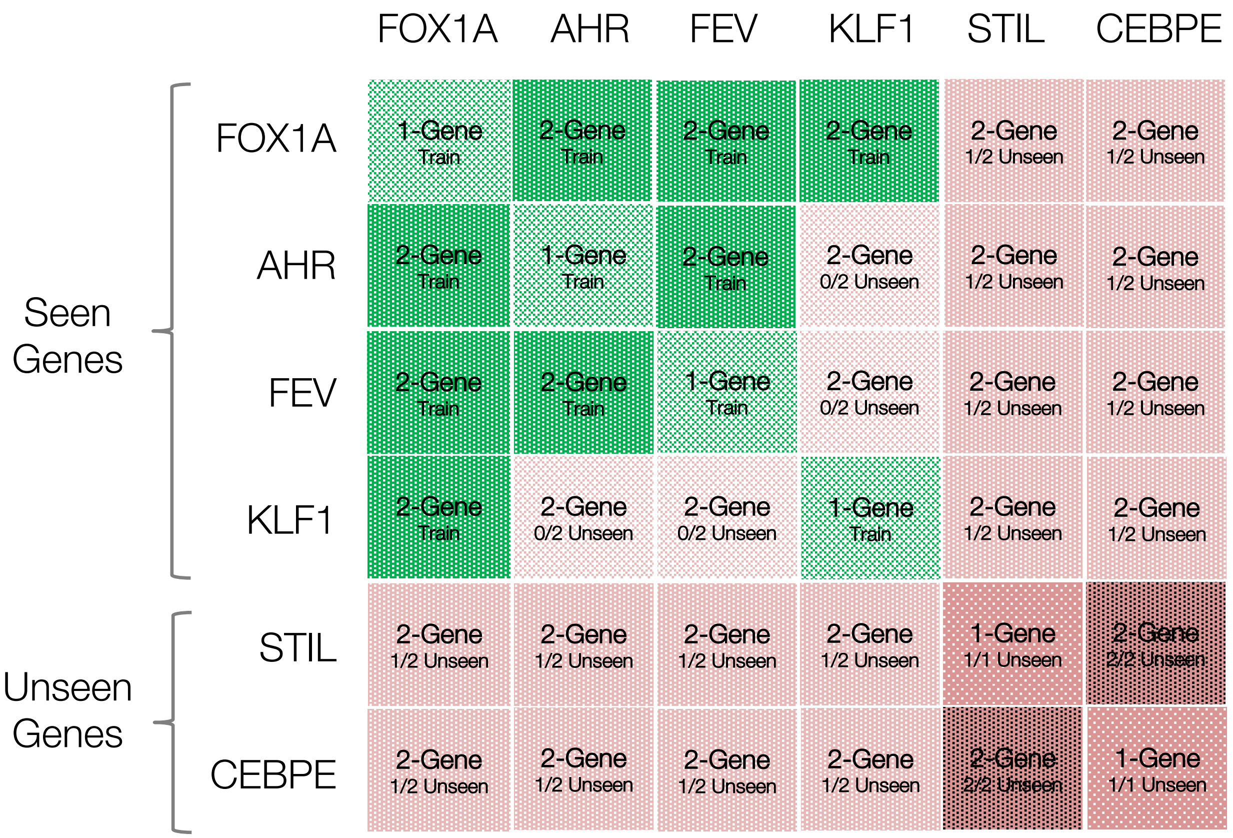

Supplementary Fig. 4: Data split matrix A sample data split illustration to describe how the different perturbation categories were defined on the basis of training set composition. Genes that were *seen* experimentally perturbed in the training data and *unseen* genes are marked on the vertical axis. 1-gene perturbations of seen genes were included in the training set (**1-Gene, Train**). 1-gene perturbations of unseen genes were included in the test set (**1-Gene, 1/1 Unseen**). 2-gene combinatorial perturbations with one gene unseen were included in the test set (**2-Gene, 1/2 Unseen**) as were those with two genes unseen (**2-Gene, 0/2 Unseen**). 2-gene combinatorial perturbations with both genes seen were randomly split between train (**2-Gene, Train**) and test (**2-Gene, 2/2 Unseen**).

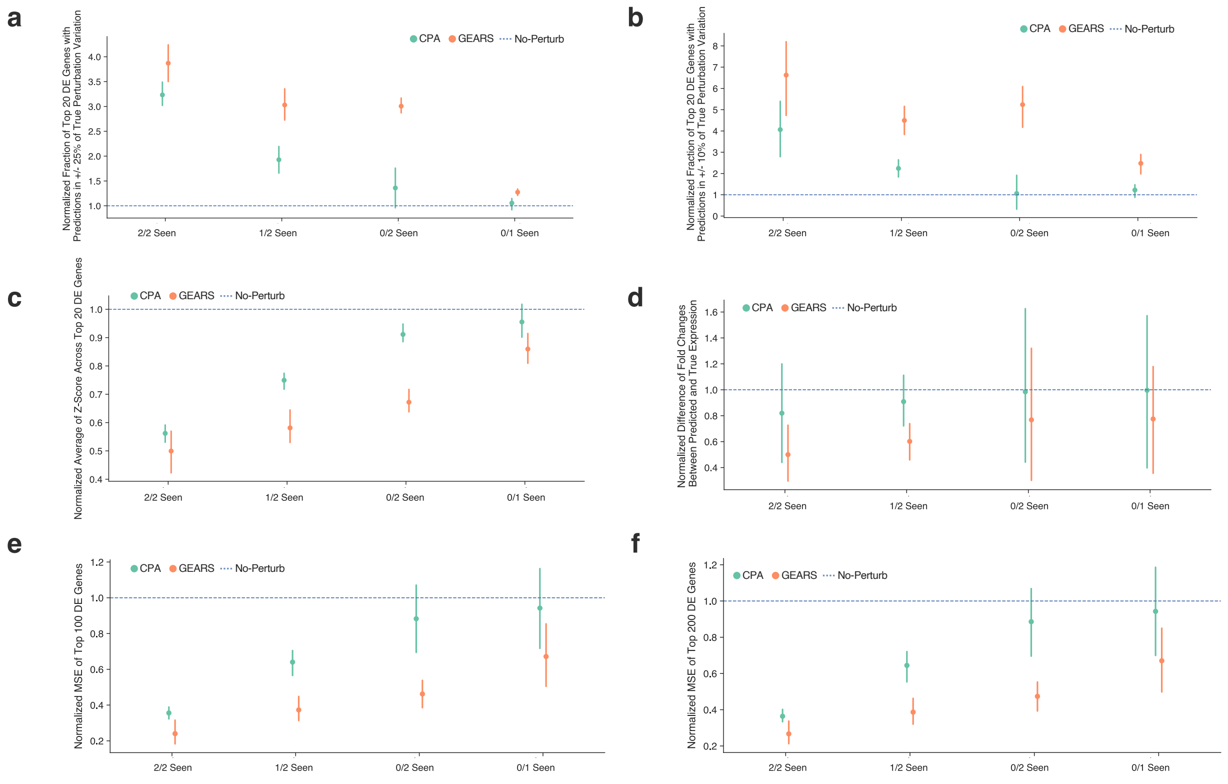

Supplementary Fig. 5: Subgroup analysis of additional evaluation metrics for predicting post-perturbation expression. (a) Fraction of top 20 differentially expressed genes with predictions fall in +/- 10% of true perturbation variation. **(b)** Fraction of top 20 differentially expressed genes with predictions fall in +/- 25% of true perturbation variation. **(c)** Average of Z-Score across top 20 differentially expressed genes. **(d)** Difference of fold changes between predicted and true expression. **(e)** MSE of Top 100 differentially expressed genes. **(f)** MSE of Top 200 differentially expressed genes. For all panels **(a-f)** the marker indicates the mean value of the corresponding metric as measured over predictions made by models trained using 5 different training data splits (n=5). The error bars represent bootstrapped 95% CI.

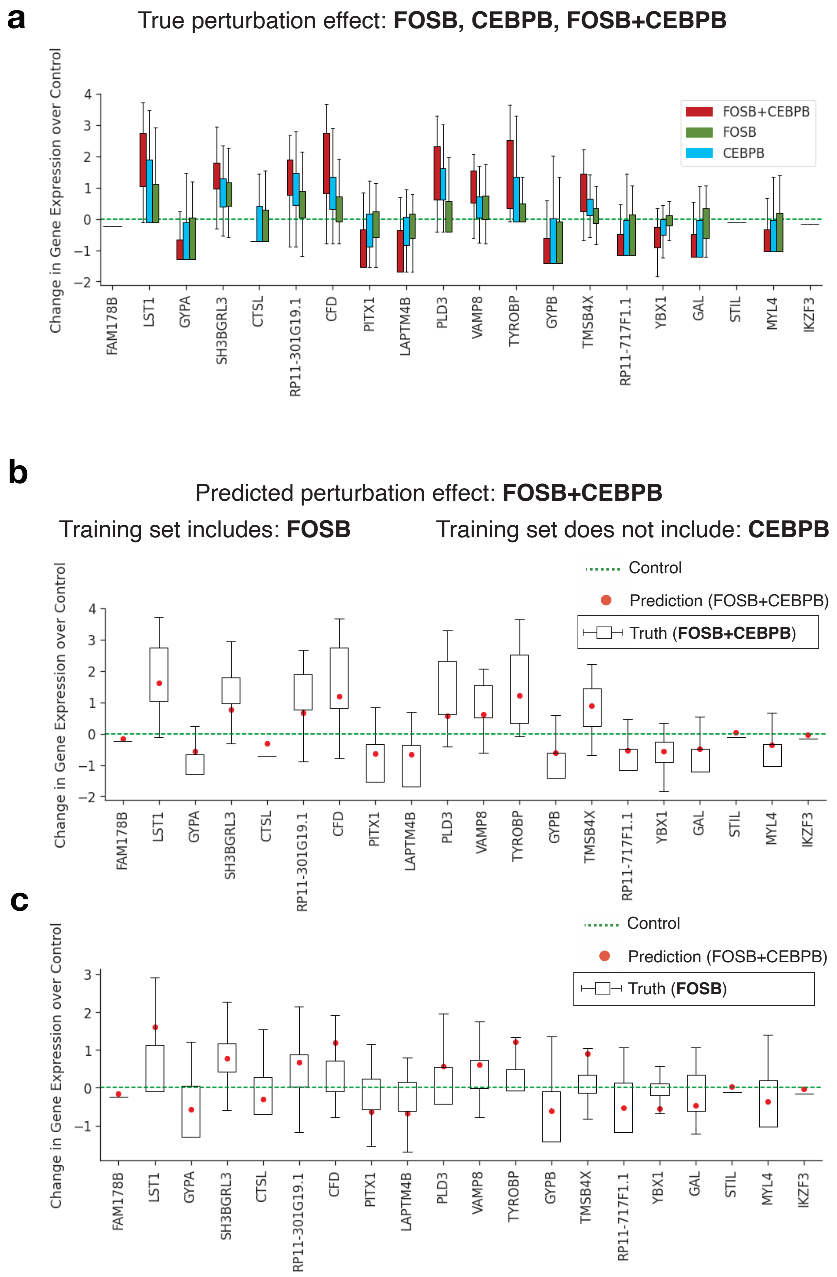

Supplementary Fig. 6: GEARS predicted post-perturbation gene expression for 2-gene combination is different from the previously seen 1-gene perturbation phenotype. The green dotted line corresponds to the mean unperturbed control expression for each gene, the boxes indicate true post-perturbation differential gene expression over control and the red symbol is the mean post-perturbation differential expression predicted by GEARS. Whiskers represent last data point within 1.5x interquartile range below the first quantile and above the third quantile. **(a)** True post-perturbation gene expression across 20 most differentially expressed genes after the combinatorial perturbation (*FOSB+CEBPB*, n=85), and the single-gene perturbations (*FOSB*, n=240), (*CEBPB*, n=502). **(b)** Predicted gene expression across 20 most differentially expressed genes after a combinatorial perturbation (*FOSB+CEBPB*). Only *FOSB* has been seen experimentally perturbed at the time of training (1 Unseen of 2) Boxplot correspond to true values (n=85). **(c)** In this case, the predictions are for the perturbation of (*FOSB+CEBPB*) but the boxplot corresponds to the effect of just perturbing *FOSB* (n=502).

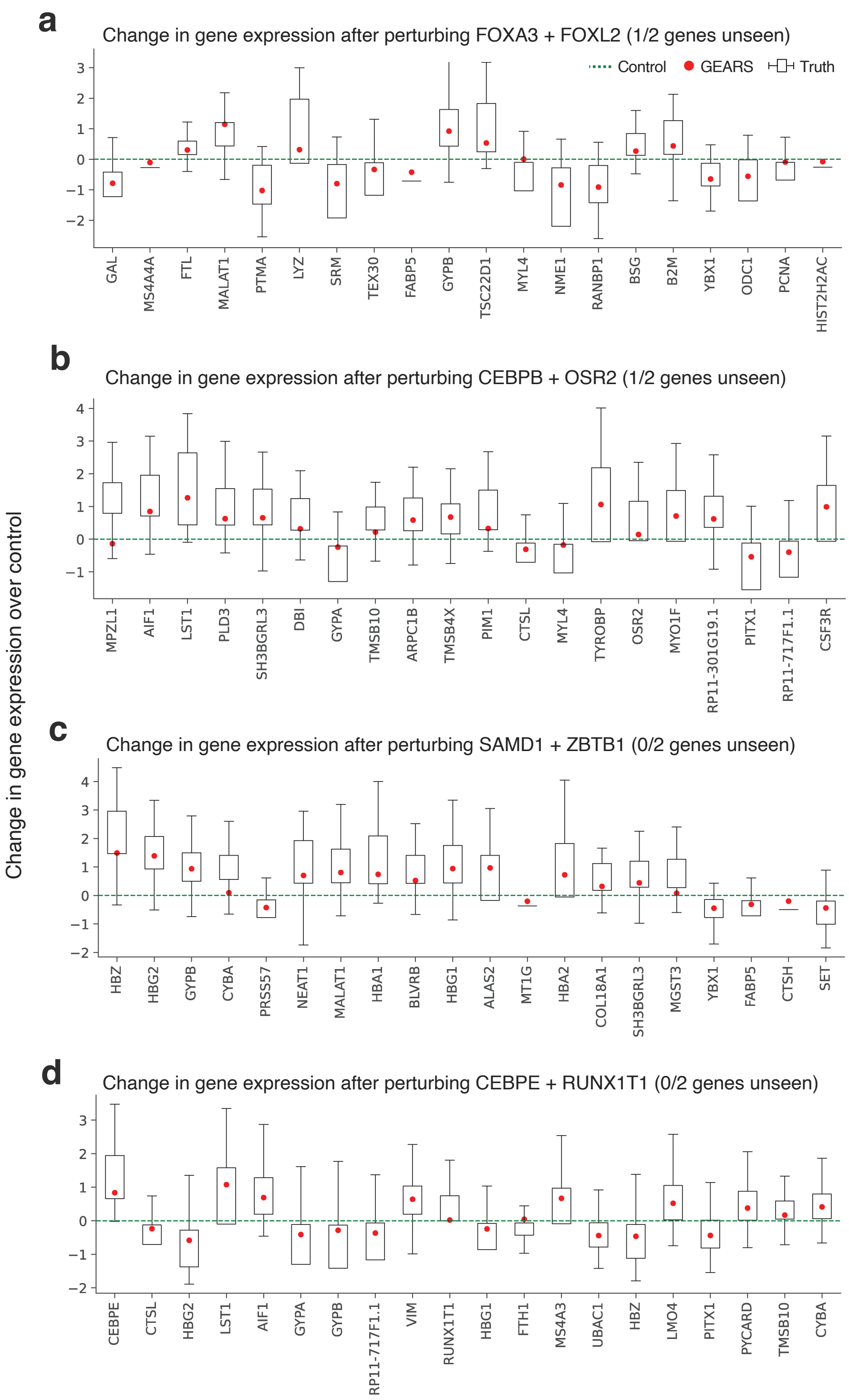

Supplementary Fig. 7: Examples of predicted gene expression across 20 most differentially expressed genes after combinatorial perturbation. Red symbol indicates GEARS prediction of mean post perturbation gene expression. **(a)** Change in gene expression after perturbing FOXA3+FOXL2 (Truth n=113). **(b)** Change in gene expression after perturbing CEBPB+MAPK1 (n=337). **(c)** Change in gene expression after perturbing FEV+MAP7D1 (n=246). **(d)** Change in gene expression after perturbing SAMD1+ZBTB1 (n=164).**(e)** Change in gene expression after perturbing ETS2+IKZF3 (n=388). **(f)** Change in gene expression after perturbing CEBPE+RUNX1T1 (n=1030). Whiskers represent last data point within 1.5x interquartile range below the first quantile and above the third quantile.

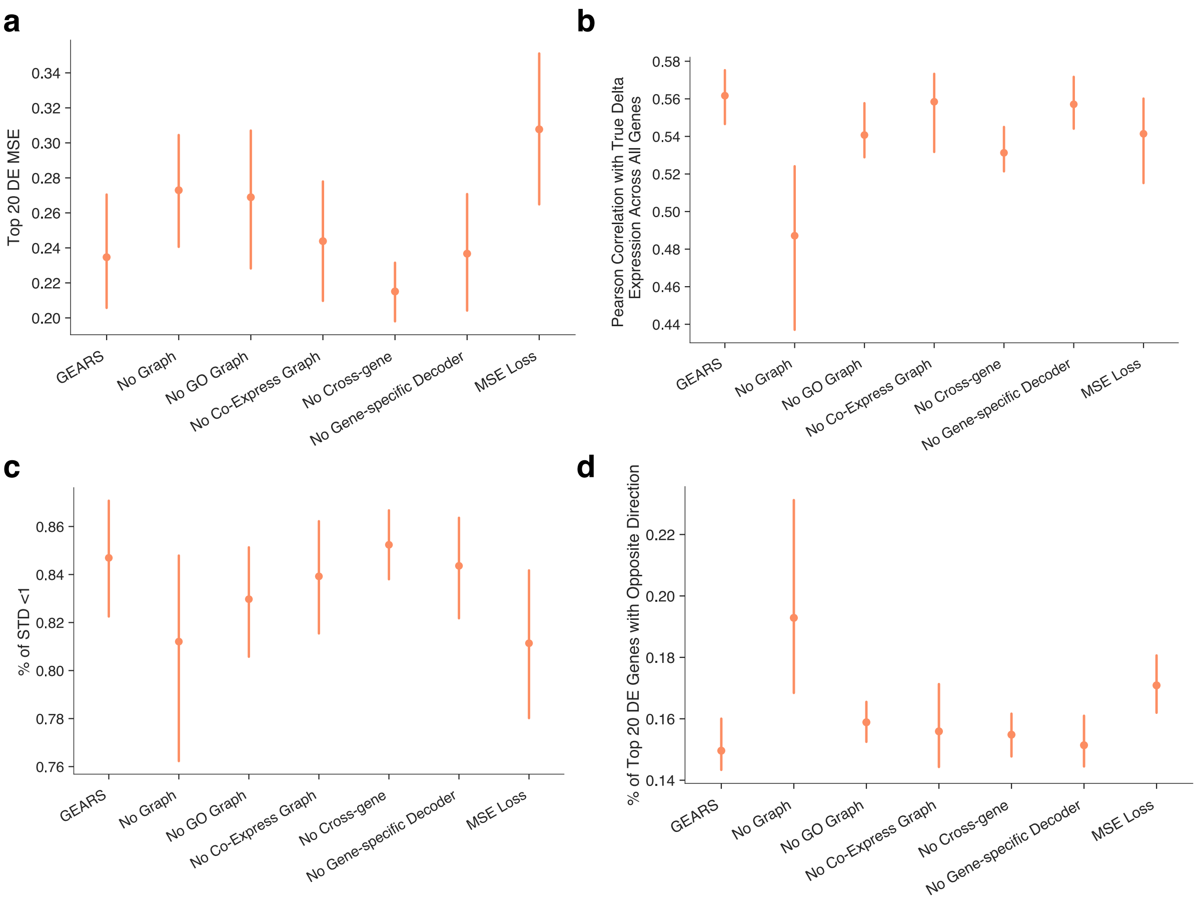

Supplementary Fig. 8: Model Ablation performance across metrics. Evaluation of importance of each component of GEARS by testing the performance after removing individual component. ”No Graph” removes both the gene ontology graph and co-expression graph; ”No GO Graph” removes the gene ontology graph; ”No Co-Express Graph” removes the co-expression graph; ”No Cross-gene” removes the cross-gene MLP layer; ”No Gene-specific Decoder” removes the gene specific decoder MLP and uses a shared MLP instead; ”MSE Loss” switches from the auto-focus loss to the regular L2 loss. **(a)** Model ablation in MSE of top 20 most differentially expressed genes. **(b)** Model ablation in pearson correlation between the true mean post-perturbation differential expression over control for across all genes and that which is predicted for the same. **(c)** Percentage of top 20 differentially expressed genes that fall within one standard deviation of the true post-perturbation gene expression distribution. **(d)** Percentage of top 20 differentially expressed genes that have the opposite direction as compared to the true post-perturbation gene expression direction. For all panels **(a-d)** the marker indicates the mean value of the corresponding metric as measured over predictions made by models trained using 5 different training data splits (n=5). The error bars represent bootstrapped 95% CI.

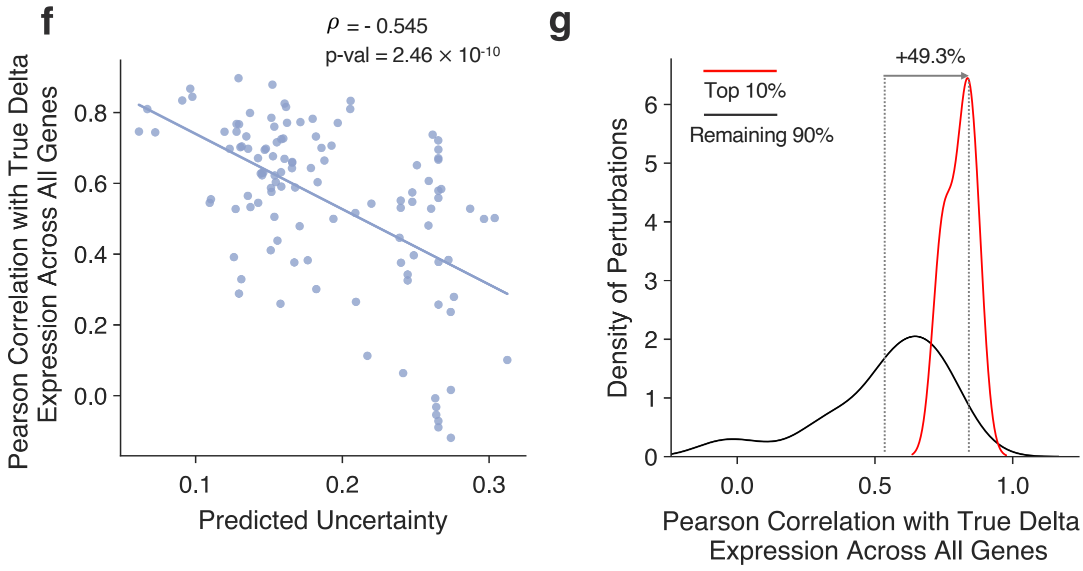

Supplementary Fig. 9: Variation in model performance between predictions with low uncertainty and others. **(a)** Scatterplot showing correlation between model predictive performance (y-axis) and predicted uncertainty (x-axis) (n=107). The p-value was computed using a two-sided test whose null hypothesis is that the distributions underlying the samples are uncorrelated. **(b)** The x-axis measures the Pearson correlation of predicted post-perturbation differential expression values over control and true post-perturbation differential expression over control over all genes. The red distribution (n=11) corresponds to perturbations with the lowest 10% predicted uncertainty while the black curve (n=96) corresponds to all other perturbations

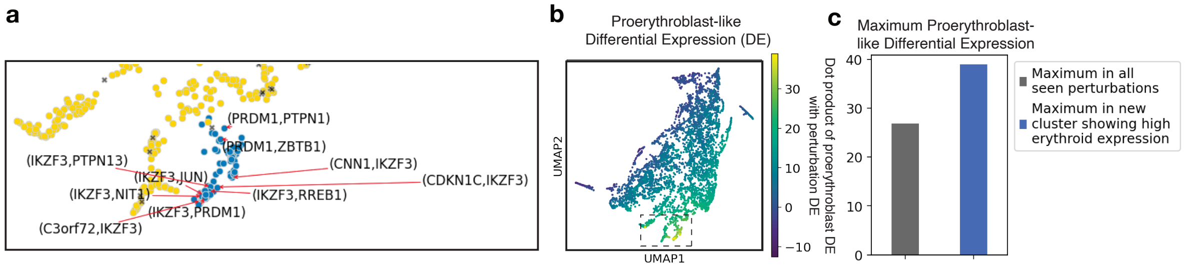

Supplementary Fig. 10: GEARS-predicted phenotype is biologically meaningful (a) GEARS identifies a novel phenotypic cluster of 90 perturbations which displayed significantly higher erythroid marker expression. A random selection of perturbations is labelled. **(b)** Novel cluster identified by GEARS shows differential expression (DE) most similar to proerythroblast-like DE. Color bar measures the dot product between the DE corresponding to the transition from hematopoietic progenitor cells to proerythroblasts (from Tabula Sapiens) and that for the transition from unperturbed controls to each perturbation outcome. **(c)** Maximum proerythroblast-like DE observed for perturbations in the novel cluster is much higher than that observed for any post-perturbation phenotype seen experimentally at the time of training (n=1).

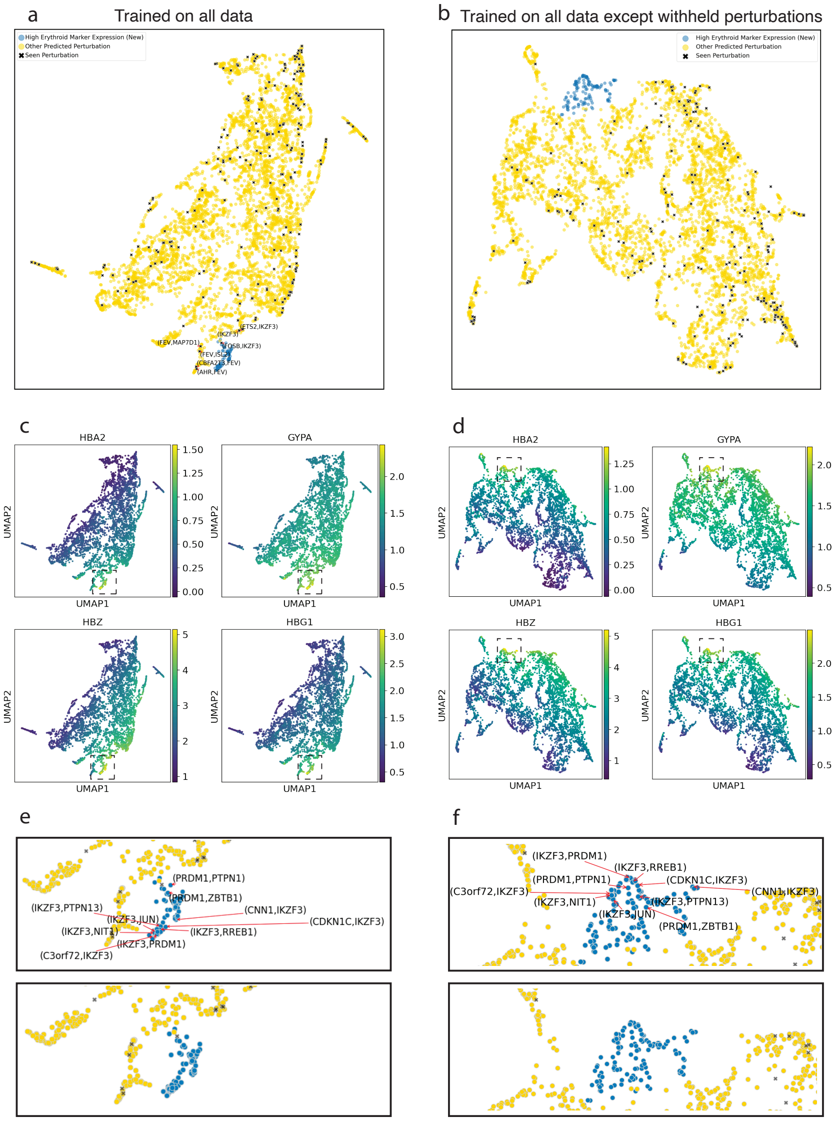

Supplementary Fig. 11: Prediction of novel phenotype robust to witholding of phenotypically similar data points. (a) GEARS predicted post-perturbation gene expression for all 5,151 pairwise combinations (and 1-gene perturbations) of the 102 single genes seen experimentally perturbed in [6]. Low-dimensional (UMAP) representation shows how predicted post-perturbation phenotypes (non-black symbols) are often novel and different from phenotypes seen experimentally (black symbols). Blue cluster indicates novel phenotype not seen during training that shows high erythroid marker expression. Labelled perturbations correspond to seven phenotypically similar perturbations that were withheld from the training set to test for model robustness. **(b)** UMAP representation of post perturbation gene expression predicted by GEARS following removal of seven training data points observed to be most similar to the detected novel phenotype in (a). **(c)** Expression of known erythroid marker genes in original UMAP space. Highest expression is observed in novel phenotypic cluster. **(d)** Highest expression is still observed in novel phenotypic cluster in new UMAP space following model training after withholding data. **(e,f)** Randomly chosen, common set of post-perturbation phenotypes that are found within the novel phenotypic cluster in both UMAP spaces. **(f)** In the new UMAP space, the cluster identified does not contain any perturbation previously seen experimentally perturbed. All plots were generated using default parameters from the UMAP package.

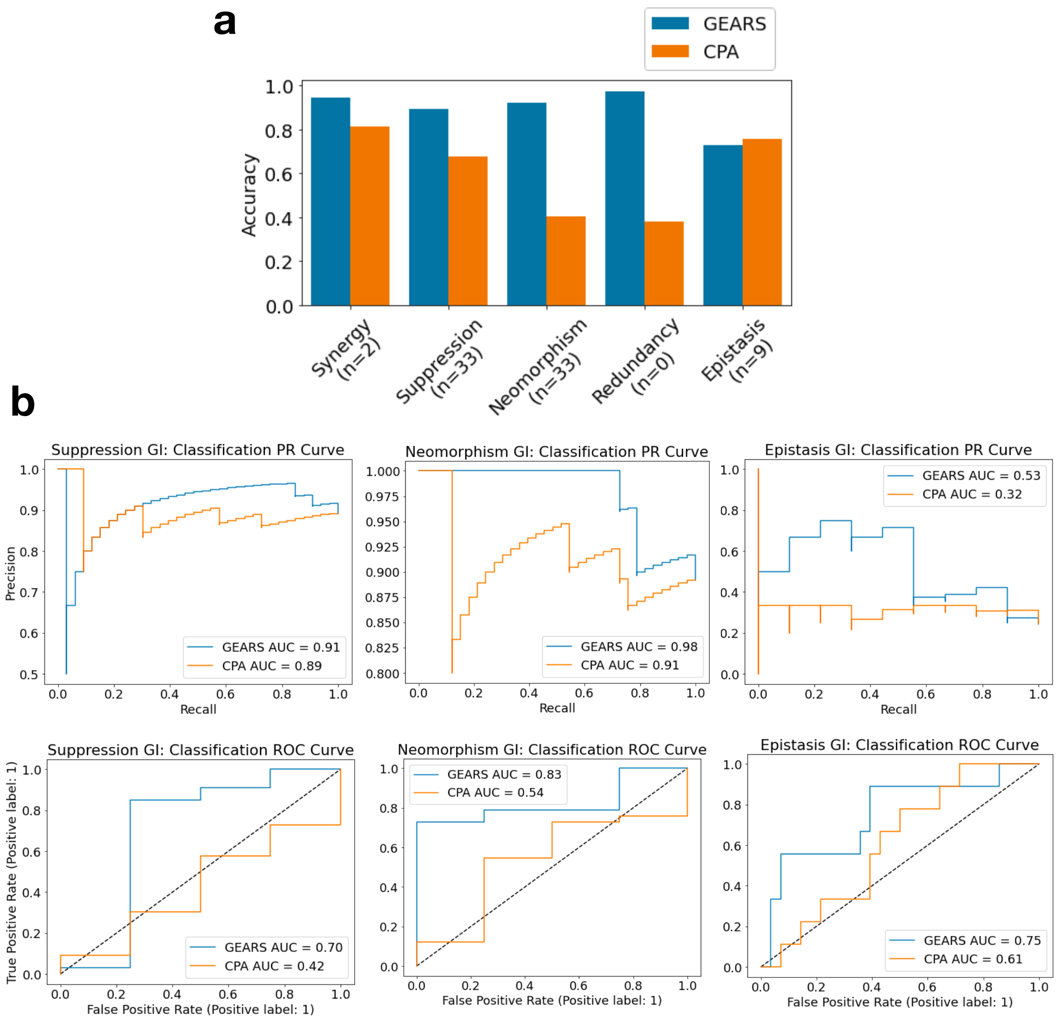

Supplementary Fig. 12: Performance evaluation of GEARS on predicting genetic interactions in [24]. **(a)** Classification accuracy for different GI subtypes. Binary classification was performed for each GI sub-type using GI scores for all 37 2-gene combinatorial perturbations in [24] (Methods). GI scores compare 2-gene combinatorial post-perturbation gene expression with that expected using a simple linear combination to measure non-linear interaction effects. **(b)** Precision-recall curves and receiver operating characteristic (ROC) curves for each genetic interaction. Legends include area under each curve (AUC). Curves not shown for synergy and redundancy because they are not meaningful for very few positive examples.

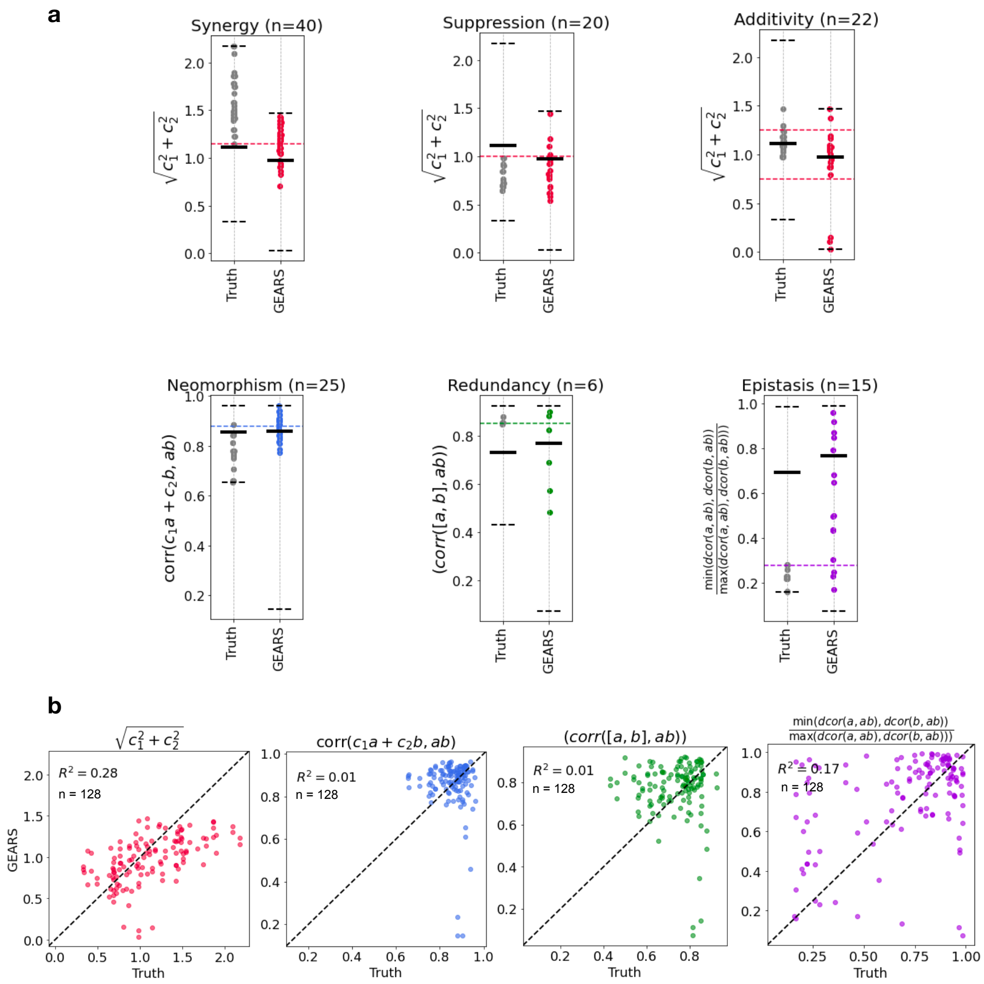

Supplementary Fig. 13: Model performance at predicting GI scores when one of the genes in a combination has not been experimentally perturbed Each combination was predicted by GEARS twice, each time holding out in the test set one of the genes in the combination (i.e. GEARS does not see any experimental perturbation containing that gene during training). The two predictions of the same combination were treated as distinct since the model used different training sets to make the predictions. Model uncertainty was used to filter approximately half of all predictions that had lower uncertainty (Supplementary Note 23). **(a)** Each plot in the panel corresponds to predicted or true GI scores for the set of combinatorial perturbations that were defined as expressing a specific GI subtype phenotype in [6]. The gray dots correspond to GI scores computed using true post-perturbation gene expression. The red dots correspond to GI scores computed using predicted post-perturbation gene expression from GEARS. GEARS was trained on all the data from [6] while only holding out all combinations that contained one specific gene, making it a novel unseen gene at the time of prediction. The metrics on the y-axis correspond to different GI scores and the dotted lines indicate the previously defined thresholds for determining if a combination is exhibiting a specific GI subtype phenotype. The black dashed lines represents the minimum and maximum of all 128 values and the black solid line represents the mean **(b)** Scatter plots of GI scores for 2-gene combinatorial perturbations in [6] that had low prediction uncertainty. The x-axis shows GI scores computed using true post-perturbation gene expression and the y-axis shows scores predicted using predicted post-perturbation gene expression. The top row shows predictions made by GEARS. R^2 refers to the coefficient of determination.

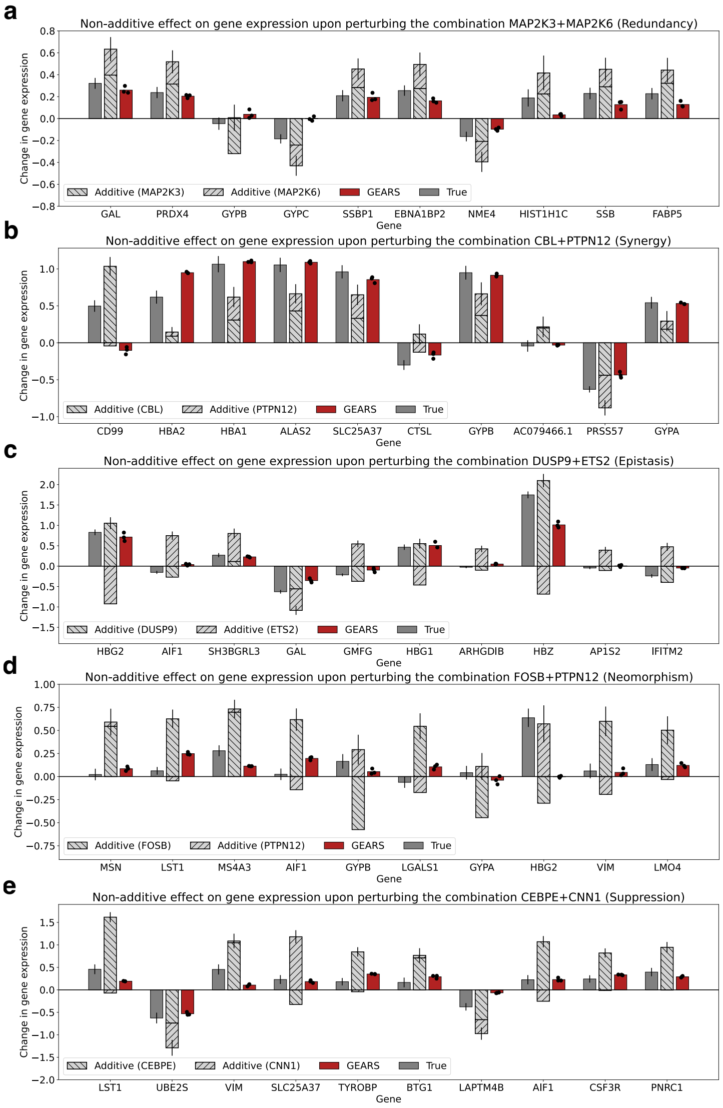

Supplementary Fig. 14: GEARS predicts non-additive combinatorial effects across all GI subtypes Each panels shows a change in gene expression over unperturbed control after perturbing a combination of genes corresponding to a specific GI subtype. The gray bars show the true mean post-perturbation gene expression change over unperturbed control for a particular gene. The hatched yellow bars show the true mean post-perturbation gene expression for each of the two single-gene perturbations performed individually. The naive additive model assumes that the effect of the combination is just the sum of the two known single-gene perturbation outcomes. The red bar indicates the prediction made by GEARS. The genes on the x-axis are those with the largest difference between true mean post-perturbation expression following combinatorial perturbation and the additive prediction for that combination. The different GI subtypes considered are (n numbers are for true data): **(a)** Redundancy (n: Combo=458, *MAP2K3*=458, *MAP2K6*=363) **(b)** Synergy (n: Combo=257, *CBL*=538, *PTPN12*=194) **(c)** Epistasis (n: Combo=698, *DUSP9*=662, *ETS2*=375) **(d)** Neomorphism (n: Combo=291, *FOSB*=502, *PTPN12*=194) **(e)** Suppression (n: Combo=194, *CEBPE*=473, *CNN1*=236). For all panels: error bars correspond to 95% CI; the bar represents the mean; n=3 for all GEARS' predictions.

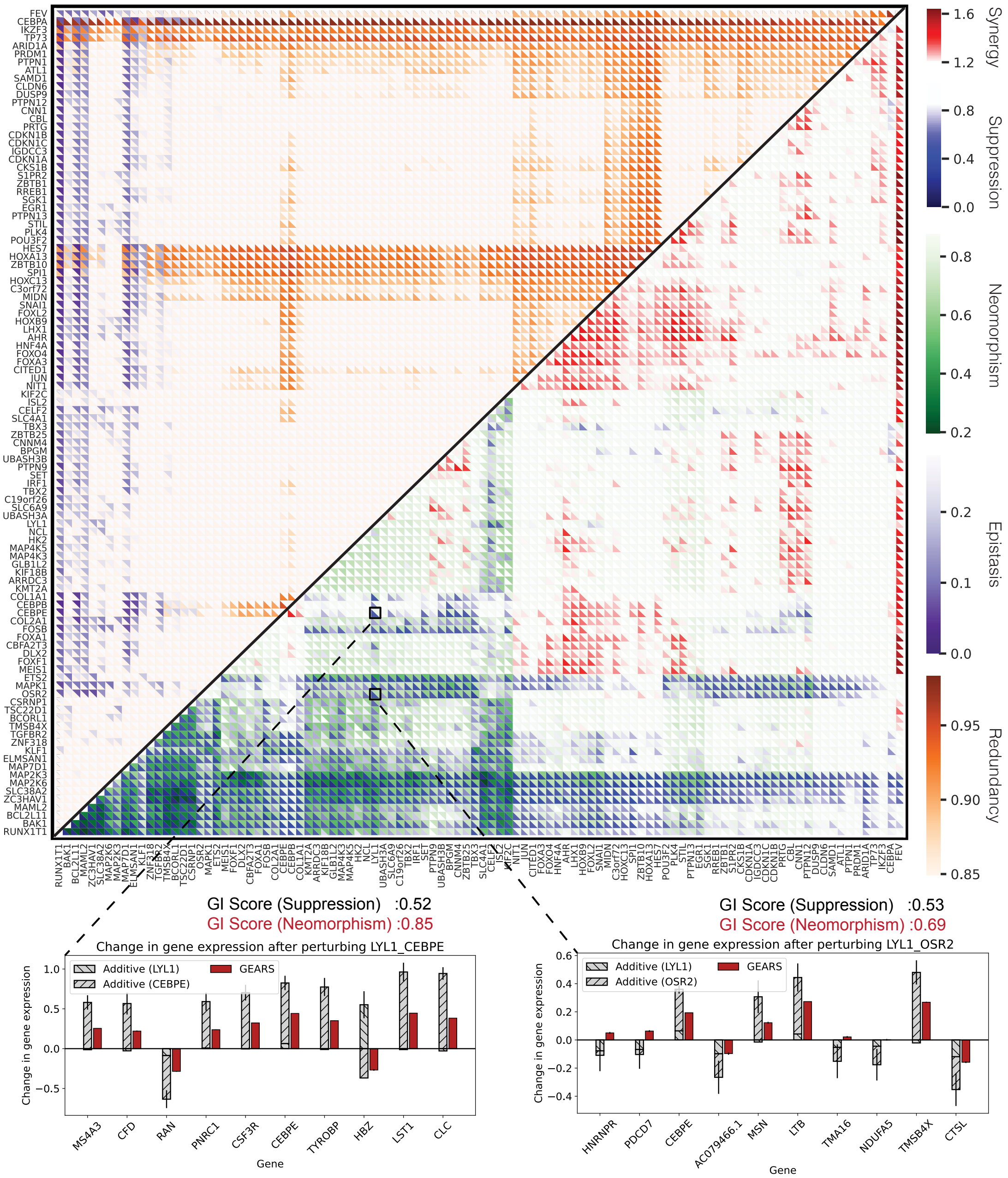

Supplementary Fig. 15: Illustration of how the multi-dimensional GI map can capture significant differences in transcriptional response: Even though *LYL1+CEBPE* and *LYL1+OSR2* are predicted to produce the same GI score for suppression, the GI score for neomorphism indicates that the predicted outcomes are different. GEARS predicts that *LYL1+OSR2* shows considerable variability in the direction and magnitude of predicted gene expression compared to an additive model. On the other hand, *LYL1+CEBPE* shows a consistent suppressive phenotype. This difference would not be detected by a conventional synergy-based GI map. Bars correspond to the mean and error bars correspond to 95% CI (*LYL1*: n=360, *CEBPE*: n= 473, *OSR2*: n= 407). GEARS predictions for both panels correspond to 100 different samples from the same trained model (n=100). While the neomorphic phenotype in this case has not been experimentally validated, both combinations were validated as suppressive using experimental data from a cell fitness screen (Cell fitness values: 0.100, 0.104 $>$ 0.099 (95th percentile of cell fitness distribution))

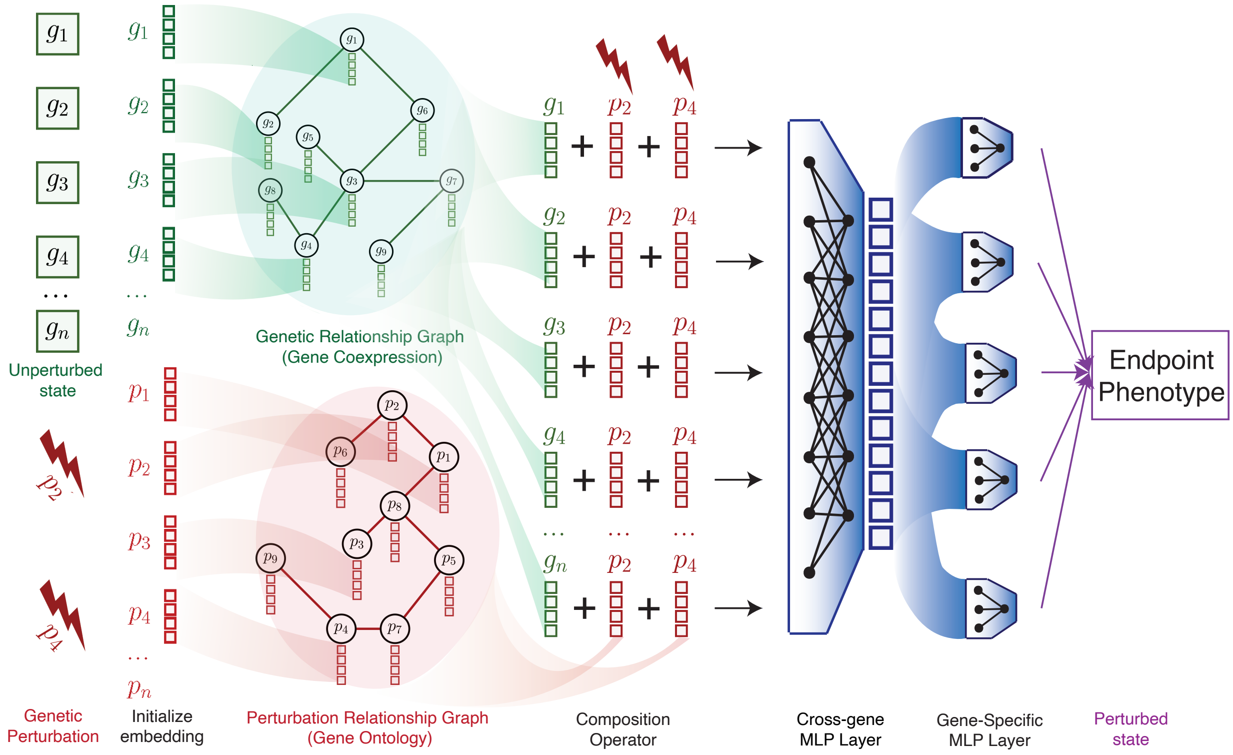

Supplementary Fig. 16: GEARS architecture can be repurposed for predicting endpoint phenotypic effects Standard GEARS architecture as in Main Figure 1, up until the last layer where an additional output layer is added to predict endpoint phenotypic effects such as cell fitness. All other input and output characteristics remain the same and the same network can still be used for predicting post-perturbation gene expression values.

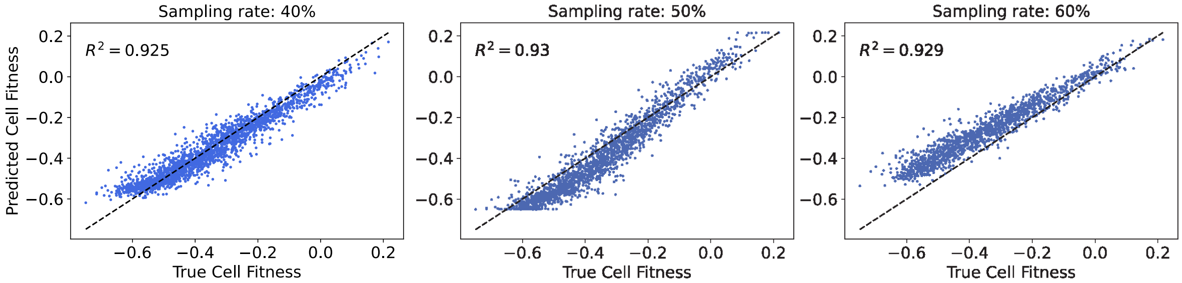

Author	Cell Type	Sampling Rate	# Test Perturbations	R^2 (Cell Fitness Prediction)
Norman 2019 [6]	K562	40	2303	0.925
Norman 2019 [6]	K562	50	1917	0.930
Norman 2019 [6]	K562	60	1527	0.929
Horlbeck 2018 [15]	K562	35	65362	0.640
Horlbeck 2018 [15]	Jurkat	35	61934	0.890

Supplementary Fig. 17: GEARS accurately predicts cell fitness scores following combinatorial perturbation Scatter plots showing GEARS predicted cell fitness score compared to true fitness score for all 2-gene combinatorial perturbations in [6]. Different plots correspond to different sampling rates for training data. For example: A sampling rate of 40% implies that 40% of the dataset was randomly sampled to form the training dataset. The table shows GEARS performs consistently well on this task across datasets and cell types.

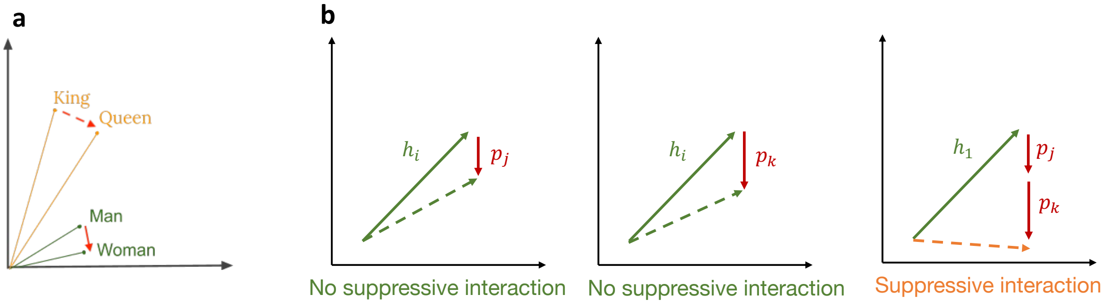

Supplementary Fig. 18: Relationship between word embeddings in NLP models and gene perturbation embeddings in GEARS: **(a)** Word embeddings can be learnt such that translation within the latent space is meaningful. In this case the vector in latent space representing the change from Man to Woman can be applied to the vector for King to obtain Queen. **(b)** Similarly, the latent space can also be made meaningful for gene embeddings. In this example a gene embedding vector with a negative slop shows a suppressive effect on its expression. Each single-gene perturbation in this case is not able to cause this effect but the combination does so.

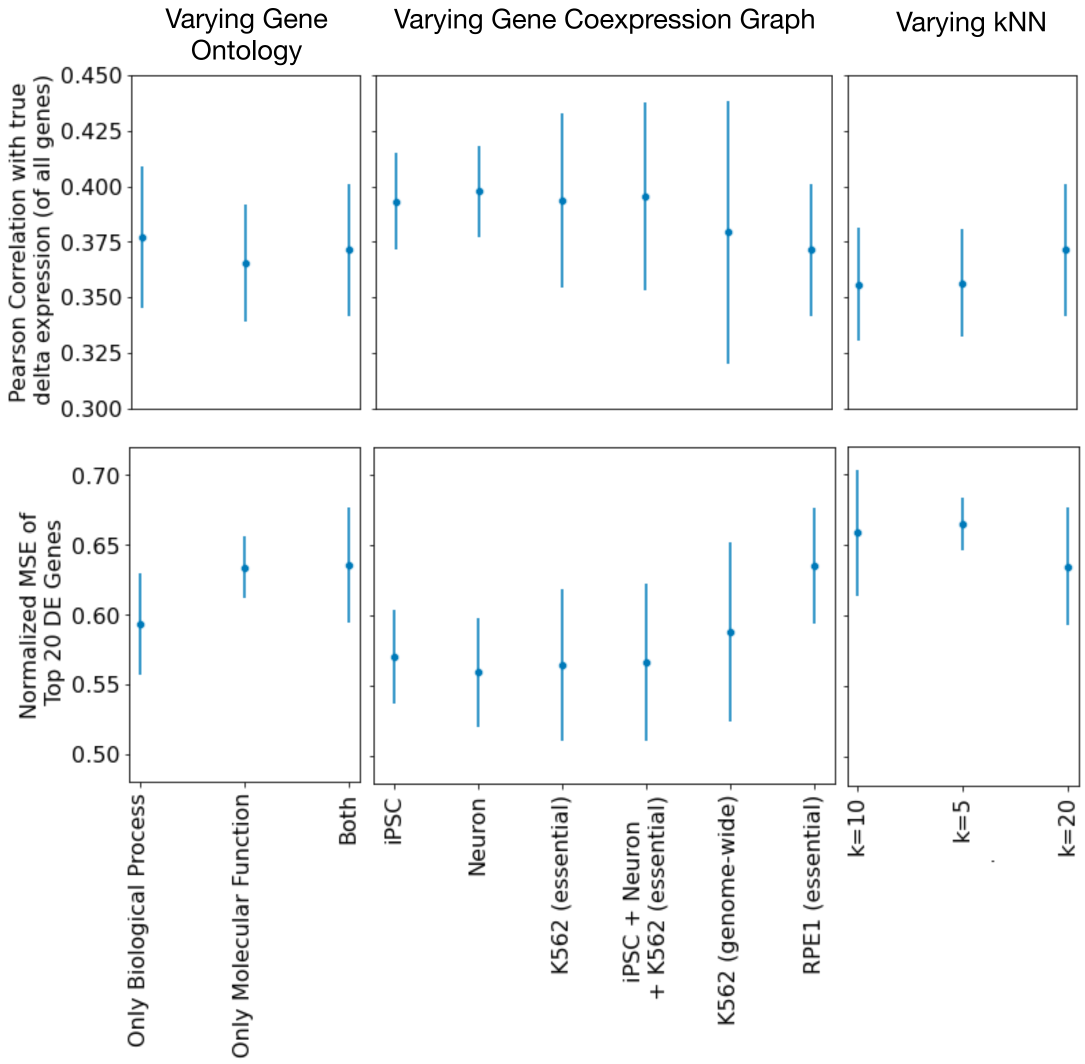

Supplementary Fig. 19: Impact of varying prior knowledge on GEARS' performance: **The left panels** look at the effect of varying the information being used from Gene Ontoloty. **The middle panels** study the effect of varying the gene co-expression graph by deriving it from gene expression datasets belonging to different cell types. **The right panels** look at the effect of varying the connectivity of the Gene Ontology graph by modifying the number of nearest neighbors that are retained when filtering edges. **The top panels** measures the Pearson correlation between the change in gene expression following perturbation predicted by GEARS to that observed in the true post-perturbation data, across all genes. **The bottom panels** looks at the mean squared error in predicted post-perturbation gene expression predicted by GEARS for the top-20 most differentially expressed genes. For all panels, markers correspond to the mean, error bars correspond to 95% CI computed over predictions made by 5 models trained using different data splits (n=5).

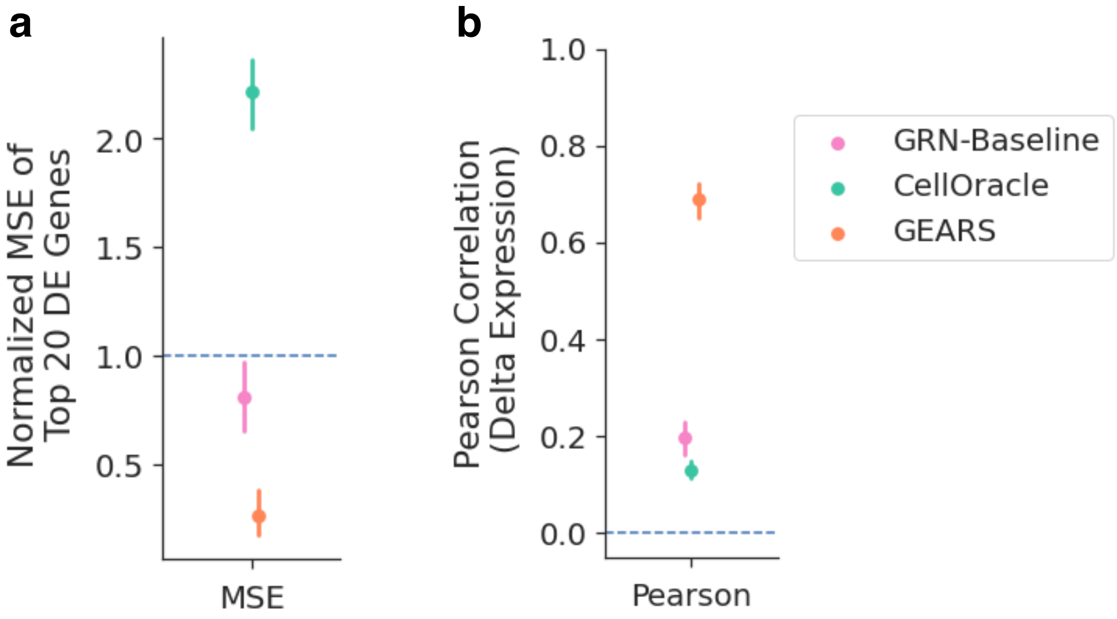

Supplementary Fig. 20: Performance comparison of GRN-Baseline, CellOracle and GEARS on the subset of perturbations that could be predicted by all three models using both 1-gene and 2-gene perturbation data from Norman et al. [6]. Metrics used: **(a)** Mean Square Error of top 20 differentially expressed genes normalized to the value predicted by the baseline model that predicts no perturbation effect **(b)** Pearson correlation between the change in gene expression for all genes following perturbation as predicted by GEARS and true change in post-perturbation gene expression. Horizontal line corresponds to no prediction made by baseline that predicts no perturbation effect. For all panels, markers correspond to the mean, error bars correspond to 95% CI computed over predictions made by 5 models trained using different data splits (n=5).

Supplementary References

1. Szklarczyk, D. *et al.* String v11: protein–protein association networks with increased coverage, supporting functional discovery in genome-wide experimental datasets. *Nucleic Acids Research* **47**, D607–D613 (2019).

2. Wainberg, M. *et al.* A genome-wide atlas of co-essential modules assigns function to uncharacterized genes. *Nature Genetics* **53**, 638–649 (2021).

3. Replogle, J. M. *et al.* Mapping information-rich genotype-phenotype landscapes with genome-scale perturb-seq. *Cell* (2022).

4. Thomas, P. D. The gene ontology and the meaning of biological function. In *The gene ontology handbook*, 15–24 (Humana Press, New York, NY, 2017).

5. Lotfollahi, M. *et al.* Learning interpretable cellular responses to complex perturbations in high-throughput screens. *bioRxiv* (2021).

6. Norman, T. M. *et al.* Exploring genetic interaction manifolds constructed from rich single-cell phenotypes. *Science* **365**, 786–793 (2019).

7. Aibar, S. *et al.* Scenic: single-cell regulatory network inference and clustering. *Nature Methods* **14**, 1083–1086 (2017).

8. Kamimoto, K. *et al.* Dissecting cell identity via network inference and in silico gene perturbation. *Nature* 1–10 (2023).

9. Yuan, B. *et al.* Cellbox: interpretable machine learning for perturbation biology with application to the design of cancer combination therapy. *Cell systems* **12**, 128–140 (2021).

10. Friedman, N., Linial, M., Nachman, I. & Pe'er, D. Using bayesian networks to analyze expression data. *Journal of Computational Biology* **7**, 601–620 (2000).

11. Pratapa, A., Jalihal, A. P., Law, J. N., Bharadwaj, A. & Murali, T. Benchmarking algorithms for gene regulatory network inference from single-cell transcriptomic data. *Nature Methods* **17**, 147–154 (2020).

12. Kamimoto, K. Celloracle simulation tutorial (2021). [Accessed: April 27, 2023].

13. Wolf, F. A., Angerer, P. & Theis, F. J. Scanpy: large-scale single-cell gene expression data analysis. *Genome biology* **19**, 1–5 (2018).

14. www.github.com/theislab/diffxpy: Differential expression analysis for single-cell rna-seq data.

15. Horlbeck, M. A. *et al.* Mapping the genetic landscape of human cells. *Cell* **174**, 953–967 (2018).

16. Li, L., Jamieson, K., DeSalvo, G., Rostamizadeh, A. & Talwalkar, A. Hyperband: A novel bandit-based approach to hyperparameter optimization. *The Journal of Machine Learning Research* **18**, 6765–6816 (2017).

17. Kipf, T. N. & Welling, M. Semi-supervised classification with graph convolutional networks. *ICLR* (2017).

18. Veličković, P. *et al.* Graph attention networks. *ICLR* (2018).

19. Wu, F. *et al.* Simplifying graph convolutional networks. In *ICML*, 6861–6871 (2019).

20. Jost, M. *et al.* Titrating gene expression using libraries of systematically attenuated crispr guide rnas. *Nature biotechnology* **38**, 355–364 (2020).

21. Dixit, A. *et al.* Perturb-seq: dissecting molecular circuits with scalable single-cell rna profiling of pooled genetic screens. *Cell* **167**, 1853–1866 (2016).

22. Tian, R. *et al.* Crispr interference-based platform for multimodal genetic screens in human ipsc-derived neurons. *Neuron* **104**, 239–255 (2019).

23. Adamson, B. *et al.* A multiplexed single-cell crispr screening platform enables systematic dissection of the unfolded protein response. *Cell* **167**, 1867–1882 (2016).

24. Replogle, J. M. *et al.* Combinatorial single-cell crispr screens by direct guide rna capture and targeted sequencing. *Nature biotechnology* **38**, 954–961 (2020).
