# Supplementary material for: Predicting transcriptional outcomes of novel multigene perturbations with GEARS
Source: Nat Biotechnol. 2023 Aug 17;42(6):927–35. doi: 10.1038/s41587-023-01905-6 (PMC11180609; doi:10.1038/s41587-023-01905-6)
Supplement: Supplementary file 2 — Reporting Summary [file 41587_2023_1905_MOESM2_ESM.pdf]

nature research

Corresponding author(s): Jure Leskovec

Last updated by author(s): May 24, 2023

# Reporting Summary

Nature Research wishes to improve the reproducibility of the work that we publish. This form provides structure for consistency and transparency in reporting. For further information on Nature Research policies, see our Editorial Policies and the Editorial Policy Checklist.

## Statistics

For all statistical analyses, confirm that the following items are present in the figure legend, table legend, main text, or Methods section.

| n/a | Confirmed | |
|---|---|---|
| ☐ | ☒ | The exact sample size (*n*) for each experimental group/condition, given as a discrete number and unit of measurement |
| ☒ | ☐ | A statement on whether measurements were taken from distinct samples or whether the same sample was measured repeatedly |
| ☐ | ☒ | The statistical test(s) used AND whether they are one- or two-sided<br>*Only common tests should be described solely by name; describe more complex techniques in the Methods section.* |
| ☒ | ☐ | A description of all covariates tested |
| ☒ | ☐ | A description of any assumptions or corrections, such as tests of normality and adjustment for multiple comparisons |
| ☐ | ☒ | A full description of the statistical parameters including central tendency (e.g. means) or other basic estimates (e.g. regression coefficient) AND variation (e.g. standard deviation) or associated estimates of uncertainty (e.g. confidence intervals) |
| ☐ | ☒ | For null hypothesis testing, the test statistic (e.g. *F*, *t*, *r*) with confidence intervals, effect sizes, degrees of freedom and *P* value noted<br>*Give P values as exact values whenever suitable.* |
| ☒ | ☐ | For Bayesian analysis, information on the choice of priors and Markov chain Monte Carlo settings |
| ☒ | ☐ | For hierarchical and complex designs, identification of the appropriate level for tests and full reporting of outcomes |
| ☐ | ☒ | Estimates of effect sizes (e.g. Cohen's *d*, Pearson's *r*), indicating how they were calculated |

*Our web collection on statistics for biologists contains articles on many of the points above.*

## Software and code

Policy information about availability of computer code

| | |
|---|---|
| Data collection | NA |
| Data analysis | All model code is available at https://www.github.com/snap-stanford/GEARS. Results can be reproduced using code available at https://github.com/yhr91/gears_misc.<br>Other packages used are: CellOracle (v 0.12.0) (https://github.com/morris-lab/CellOracle), CPA (https://github.com/facebookresearch/CPA, Accessed 09/2021), pySCENIC (v 0.12.0) |

For manuscripts utilizing custom algorithms or software that are central to the research but not yet described in published literature, software must be made available to editors and reviewers. We strongly encourage code deposition in a community repository (e.g. GitHub). See the Nature Research guidelines for submitting code & software for further information.

## Data

Policy information about availability of data

All manuscripts must include a data availability statement. This statement should provide the following information, where applicable:

- Accession codes, unique identifiers, or web links for publicly available datasets
- A list of figures that have associated raw data
- A description of any restrictions on data availability

Only publicly available datasets were used:
Dixit et al., Cell (2016) GEO Accession Number GSE90063
Adamson et al., Cell (2016) GEO Accession Number GSE90546
Norman et al., Science (2019) GEO Accession Number GSE133344
Replogle et al. Cell (2022) Figshare DOI https://doi.org/10.25452/figshare.plus.20029387.v1

Jost et al. Nature Biotech. (2022) GEO Accession Number GSE132080
Tian et al. Neuron (2019) GEO Accession Number GSE124703
Replogle et al. Nature Biotech. (2020) GEO Accession Number: GSE146194
Horlbeck et al. Cell (2018) GEO Accession Number: GSE116198

There are no figures with associated raw data.
There are no restrictions on data availability

# Field-specific reporting

Please select the one below that is the best fit for your research. If you are not sure, read the appropriate sections before making your selection.

☒ Life sciences ☐ Behavioural & social sciences ☐ Ecological, evolutionary & environmental sciences

For a reference copy of the document with all sections, see nature.com/documents/nr-reporting-summary-flat.pdf

# Life sciences study design

All studies must disclose on these points even when the disclosure is negative.

| | |
|---|---|
| Sample size | In case of experimental data, we used full published datasets, so sample size was defined by the studies that generated the data and any relevant post-processing.<br>For computational experiments, we used 5 different random train-test data splits which is typical in the field (n=5). In the case of leave-one-out computational validation (e.g. for genetic interaction prediction), we ran the model 3 different times since the test set is fixed and variation between predictions is not significant (n=3) |
| Data exclusions | No data was excluded from the public datasets listed above |
| Replication | Computational experiments are all repeatable using the code provided. |
| Randomization | Not required as we were not running any new gene perturbation experiments. |
| Blinding | Not required as we were not running any new gene perturbation experiments. |

# Reporting for specific materials, systems and methods

We require information from authors about some types of materials, experimental systems and methods used in many studies. Here, indicate whether each material, system or method listed is relevant to your study. If you are not sure if a list item applies to your research, read the appropriate section before selecting a response.

## Materials & experimental systems

| n/a | Involved in the study |
|---|---|
| ☒ | ☐ Antibodies |
| ☒ | ☐ Eukaryotic cell lines |
| ☒ | ☐ Palaeontology and archaeology |
| ☒ | ☐ Animals and other organisms |
| ☒ | ☐ Human research participants |
| ☒ | ☐ Clinical data |
| ☒ | ☐ Dual use research of concern |

## Methods

| n/a | Involved in the study |
|---|---|
| ☒ | ☐ ChIP-seq |
| ☒ | ☐ Flow cytometry |
| ☒ | ☐ MRI-based neuroimaging |
